# Persistent enhancement of basolateral amygdala-dorsomedial striatum synapses causes compulsive-like behaviors in mice

**DOI:** 10.1038/s41467-023-44322-8

**Published:** 2024-01-08

**Authors:** In Bum Lee, Eugene Lee, Na-Eun Han, Marko Slavuj, Jeong Wook Hwang, Ahrim Lee, Taeyoung Sun, Yehwan Jeong, Ja-Hyun Baik, Jae-Yong Park, Se-Young Choi, Jeehyun Kwag, Bong-June Yoon

**Affiliations:** 1 Department of Life Sciences, Korea University, Seoul, 02841 Republic of Korea; 2 Department of Brain and Cognitive Engineering, Korea University, Seoul, 02841 Republic of Korea; 3 School of Biosystems and Biomedical Sciences, College of Health Sciences, Korea University, Seoul, 02841 Republic of Korea; 4 Department of Physiology, Dental Research Institute, Seoul National University School of Dentistry, Seoul, 03080 Republic of Korea; 5 Department of Brain and Cognitive Sciences, Seoul National University, Seoul, 08826 Republic of Korea

**Keywords:** Neural circuits, Striatum

## Abstract

Compulsive behaviors are observed in a range of psychiatric disorders, however the neural substrates underlying the behaviors are not clearly defined. Here we show that the basolateral amygdala-dorsomedial striatum (BLA-DMS) circuit activation leads to the manifestation of compulsive-like behaviors. We revealed that the BLA neurons projecting to the DMS, mainly onto dopamine D1 receptor-expressing neurons, largely overlap with the neuronal population that responds to aversive predator stress, a widely used anxiogenic stressor. Specific optogenetic activation of the BLA-DMS circuit induced a strong anxiety response followed by compulsive grooming. Furthermore, we developed a mouse model for compulsivity displaying a wide spectrum of compulsive-like behaviors by chronically activating the BLA-DMS circuit. In these mice, persistent molecular changes at the BLA-DMS synapses observed were causally related to the compulsive-like phenotypes. Together, our study demonstrates the involvement of the BLA-DMS circuit in the emergence of enduring compulsive-like behaviors via its persistent synaptic changes.

## Abstract (web summary)

Compulsivity is associated with many psychiatric disorders including obsessive compulsive disorder. However, the neural circuits that mediate this trait are not clearly defined. Here the authors show that the amygdalostriatal circuit plays a critical role in the pathophysiology of compulsive-like behavior.

## Introduction

Compulsive behavior consists of actions that are inappropriate to the situations without discernible connection to the overall goal^1^ and can be observed in a variety of psychiatric disorders from obsessive-compulsive disorders (OCD) to Tourette tic disorder, drug addiction, eating disorders, autism spectrum disorders and compulsive gambling^2^. It has been hypothesized that compulsivity is caused by dysregulation of response inhibition or cognitive control and an impaired balance between goal-directed behavior and habit learning^2^. While brain circuitry subserving compulsivity is not fully elucidated, studies on OCD have shown hypermetabolism or hyperactivity in brain regions comprising the cortico-striatal-thalamo-cortical (CSTC) circuit in patients with OCD^3–8^. Furthermore, a recent meta-analysis study has provided evidence for the involvement of the amygdala, a key brain area involved in fear and anxiety, in OCD^9^, which was previously controversial due to mixed findings^10–12^. The possible involvement of the amygdala in compulsive behavior implicates affective dysregulation in the emergence of compulsive behaviors^13^.

The amygdala is a primary brain structure mediating valence assignment to sensory stimuli and processing emotionally salient stimuli for further behavioral adaptations. The basolateral amygdala (BLA) and the central amygdala (CEA), two extensively studied subregions in the amygdala, participate in the amygdala functions via multiple connections to various brain areas. Although the CEA has been implicated in an interaction with the striatum^14^, whether there is a direct connectivity between the CEA and the striatum remains unclear^15,16^. In contrast, there are extensive projections from the BLA to both nucleus accumbens (NAc) and the dorsomedial striatum (DMS). The BLA-NAc circuit has been demonstrated to be involved in reward-seeking behavior or fear extinction^17–21^. However, the functional roles of the BLA to the DMS remain uncertain. The DMS is a part of limbic/associative CSTC circuit implicated in goal-directed behavior in rodents^22^. Since it is critical to take changing environment into consideration for efficient adaptive decision-making in a goal-directed action, affective states probably influence this goal-directed behavior, possibly by engaging the DMS. In fact, the hyperactive caudate nucleus (homologous to the rodent DMS) has been related to excessive habit formation in patients with OCD^23^. Furthermore, animal models showing compulsive self-grooming have demonstrated that functional and morphological alterations at synapses in corticostriatal circuits^24–26^ indicating synaptic dysfunctions within compulsivity-relevant circuits may underlie persevering actions^27^. Therefore, we hypothesized that this BLA-DMS connection might be involved in the modulation of motor behavior in response to changes in affective state and that synaptic alterations in these synapses might support long-lasting manifestation of behavioral symptoms.

Our results demonstrate that the activation of the BLA-DMS projections induced increases in anxiety and compulsive-like repetitive behaviors such as grooming and stereotypies in mice, frequently-observed behaviors in animal models with compulsivity^24–26,28,29^. We found that dopamine D1 receptor-expressing medium spiny neurons (D1-MSNs) in the DMS preferentially receive direct input from the BLA neurons that are activated under stressful conditions and that these D1-MSNs play an essential role in expressing compulsive-like behavior. Furthermore, we developed a mouse model in which the BLA-DMS input is chronically activated, which displays a variety of compulsive-like behaviors. The emergence of repetitive behaviors was associated with an increase in the ratio of alpha-amino-3-hydroxy-5-methyl-4-isoxazolepropionic acid (AMPA) to N-methyl-D-aspartate (NMDA) current ratio and a marked increase in the activation of extracellular signal-regulated kinase (ERK) at the BLA-DMS synapses. In summary, our study reveals that the BLA-DMS circuit activity mediates the stress-induced compulsive-like behaviors by recruiting D1-MSNs and that the enhanced activity of this circuit can cause persistent compulsive-like behaviors through synaptic modifications and enhanced synaptic ERK activity.

## Results

### Activating D1-MSNs in the dorsal striatum together with predator stress (PRST) exposure induces compulsive-like behaviors

**Fig. 1:**
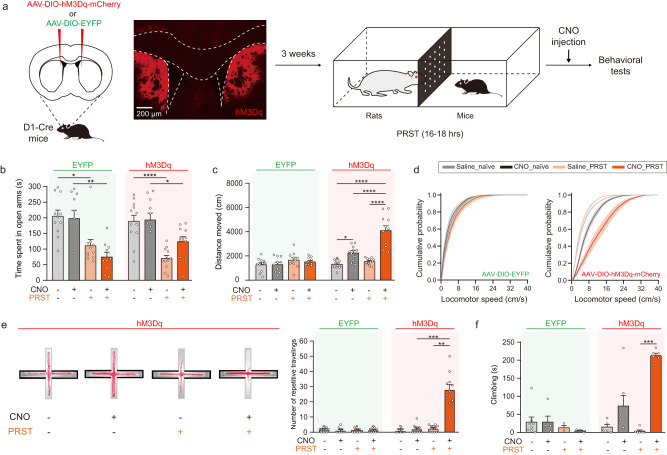
Specific activation of D1-MSNs in the dorsal striatum together with stress exposure induces repetitive behavior. **a** Schematic of the experiment. Animals were randomly assigned to either a saline or CNO (10 mg/kg) group and underwent behavioral tests without PRST exposure first. The same tests were repeated 1 week later after overnight exposure to PRST. (EYFP_Saline_, *n* = 11; EYFP_CNO_, *n* = 10; hM3Dq_Saline_, *n* = 12; hM3Dq_CNO_, *n* = 11). **b** Time spent in open arms in the EPM. Kruskal–Wallis test with post hoc Dunn’s multiple comparisons test (EYFP) (*H* = 18.59, *P* = 0.0003) and two-way ANOVA with post hoc Tukey’s test (hM3Dq) (Interaction_drug × PRST_: *F*_1, 42_ = 2.141, *P* = 0.1509, PRST effect: *F*_1, 42_ = 30.71, *P* = 1.807e^−06^) were used for data analyses. **c** Total distance traveled in the EPM. A two-way ANOVA with post hoc Tukey’s test showed a significant interaction between drug (CNO) effect and stress (PRST) (*F*_1, 42_ = 13.88, *P* = 0.0006) in the hM3Dq-injected group. **d** Cumulative probability plot of the locomotor speed (Kolmogorov–Smirnov test; Saline_PRST *vs* CNO_PRST, *P* = 1.033e^−10^; CNO_naive *vs* CNO_PRST, *P* = 3.374e^−05^). **e** Repetitive traveling pattern in PRST-exposed mice with D1-MSN activation. Left, representative movement tracks on EPM (closed arms are indicated by darker outlines). Right, quantification (Kruskal–Wallis test with post hoc Dunn’s multiple comparisons test: *H* = 27.56, *P* = 4.499e^−06^). **f** Animals subjected to EPM and exposed to PRST once were examined in the forced swim test (FST) 5–7 days after EPM test. Each of four groups of animals (EYFP_Saline_, EYFP_CNO_, hM3Dq_Saline_, hM3Dq_CNO_) were divided into two groups, Naive and PRST (EYFP_Saline_naive_, *n* = 8; EYFP_CNO_naive_, *n* = 5; EYFP_Saline_PRST_, *n* = 3; EYFP_CNO_PRST_, *n* = 3; hM3Dq_Saline_naive_, *n* = 5; hM3Dq_CNO_naive_, *n* = 7; hM3Dq_Saline_PRST_, *n* = 6; hM3Dq_CNO_PRST_, *n* = 6). Kruskal–Wallis test with post hoc Dunn’s multiple comparisons test: *H* = 17.76, *P* = 0.0005.

The basal ganglia network is functionally involved in stress-induced behaviors^30^. To assess the functional role of MSNs in anxiety-related behaviors, we injected adeno-associated virus (AAV) carrying an excitatory designer receptors exclusively activated by designer drugs (DREADD) gene, hM3Dq, whose expression is Cre recombinase-dependent, into the DMS of D1-Cre mice or D2-Cre mice (Fig. 1a). Overnight exposure to PRST induced strong anxiety as shown by a significant decrease in the time spent in the open arms of an elevated plus maze (EPM) (Fig. 1b and Supplementary Fig. 1). In addition, selective activation of D1-MSNs through clozapine-N-oxide (CNO) after PRST exposure dramatically enhanced locomotor activity (Fig. 1c, d). There was a significant interaction between the effects of drug (CNO) and stress (PRST) on total distance traveled. In contrast, D2-MSNs activation caused a general decrease in locomotor activity regardless of PRST exposure (Supplementary Fig. 1). Interestingly, the enhanced locomotor activity of the animals with D1-MSNs activation after PRST exposure consisted of highly repetitive locomotor patterns (Fig. 1e). In the forced swim test (FST), these animals also demonstrated a strong increase in active climbing behavior (Fig. 1f). These results suggested that the association of striatal activity and emotional stress might induce highly repetitive behaviors.

**Fig. 2:**
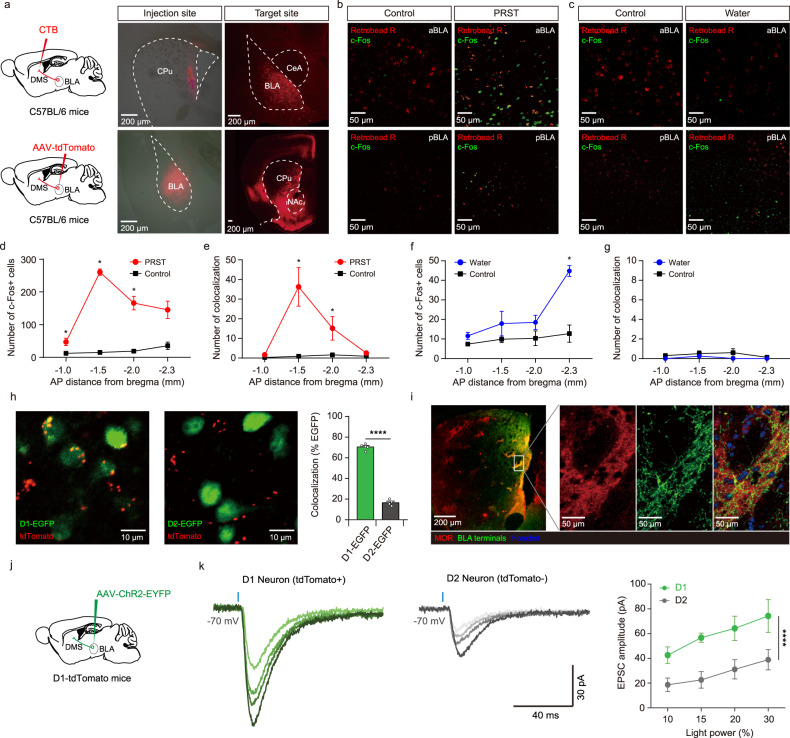
Negative-valence neurons in the BLA send axons predominantly to D1-MSNs in the DMS. **a** Schematic and representative results of retrograde (top) and anterograde (bottom) tracing of amygdalostriatal projections. The tracing experiment was repeated 5 times with similar results. **b**, **d**, **e** PRST-responsive BLA neurons send projections to the DMS. There was a significant increase in the number of c-Fos positive neurons across multiple BLA positions in PRST-exposed animals compared to control (two-sided Mann–Whitney test: −1.0, *U* = 0, *P* = 0.0286; −1.5, *U* = 0, *P* = 0.0159; −2.0, *U* = 0, *P* = 0.0159; −2.3, *U* = 0, *P* = 0.0571). However, the anterior BLA (aBLA, AP –1.5) displayed a significantly higher number of c-Fos signals than other positions and colocalization with Retrobeads R (two-sided Mann–Whitney test: −1.0, *U* = 1, *P* = 0.0571; −1.5, *U* = 0, *P* = 0.0159; −2.0, *U* = 0, *P* = 0.0159; −2.3, *U* = 1.5, *P* = 0.1429) (Control, *n* = 5; PRST, *n* = 4). **c**, **f**, **g** Reward exposure resulted in a trend of increase in c-Fos expression in the posterior amygdala (pBLA, AP –2.3). (two-sided Mann–Whitney test: −1.0, *U* = 4.5, *P* = 0.119; −1.5, *U* = 4, *P* = 0.2857; −2.0, *U* = 4, *P* = 0.1905; −2.3, *U* = 0, *P* = 0.0159). However, few c-Fos-positive cells colocalized with Retrobead R. (two-sided Mann–Whitney test: −1.0, *U* = 7.5, *P* = 0.4444; −1.5, *U* = 6, *P* = 1; −2.0, *U* = 6, *P* = 0.4444; −2.3, *U* = 7.5, *P* = 0.4444). (Control, *n* = 5; Reward, *n* = 5). **h** Target specificity of amygdalostriatal projections. AAV-tdTomato was injected into the BLA of D1-EGFP mice (left) or D2-EGFP mice (right). Representative images and quantification are shown (D1-MSNs, *n* = 657 cells from five mice; D2-MSNs, *n* = 276 cells from four mice). Two-sided unpaired *t*-test: *t* = 27.67, *df* = 7, *P* = 2.069e^−08^. **i** Patch-like patterns of the BLA-DMS inputs are colocalized with mu-opioid receptor (MOR)-positive striosomes in the DMS. Axon terminals from BLA (green), MOR signals (red), and Hoechst signals (blue) in a putative striosome structure are shown. **j** Schematic of viral injection for electrophysiological recordings using optical stimulation. **k** Left, representative traces of optical stimulation-evoked excitatory postsynaptic currents (oEPSCs) in D1-MSN neurons (tdTomato+) and D2-MSNs (tdTomato-). Blue rectangles indicate times of light stimulation (470 nm, 1 ms). Different colors (light to dark green or light gray to black) indicate oEPSC responses to different stimulation light intensities (10%, 15%, 20%, and 30% of maximal light power (15 mW)). Right, stimulus-response curves of oEPSCs recorded in D1-MSNs (*n* = 5 cells from 5 mice) and D2-MSNs (*n* = 6 cells from 6 mice). Two-way ANOVA with post hoc Tukey-Kramer test: *F*_1,42_ = 27.2232, *P* = 4.567e^−06^.

Since PRST exposure increases c-Fos expression in the amygdala^31^, we investigated whether a neural circuit connecting the amygdala and the dorsal striatum would underlie the modulation of motor behavior in the presence of an emotionally salient stimulus. Using both retrograde cell labeling and anterograde fiber tracing, we confirmed that the DMS receives BLA inputs (Fig. 2a), as previously shown by others^16,32,33^. The BLA contains distinct populations of neurons encoding negative valence or positive valence, which are genetically distinct, spatially segregated, and anatomically divergent^34,35^. We, therefore, examined whether the DMS-projecting BLA neurons encode a specific valence by evaluating the colocalization of c-Fos signals induced by positive- or negative-valence events with a retrograde dye injected in the DMS. We found a major cell population activated by PRST in the anterior part of the BLA, which showed considerable colocalization with DMS-injected retrograde dye (Fig. 2b, d, e). However, the BLA neurons responding to a water reward provided after a period of water deprivation had a different distribution pattern showing a trend of enrichment in the posterior BLA, consistent with previous studies^35,36^ (Fig. 2c, f). In contrast to PRST-responsive BLA neurons, the reward-responsive BLA neurons showed little colocalization with retrograde dye injected into the DMS (Fig. 2g). We next investigated whether these DMS-projecting BLA neurons have any target cell-type specificity. Interestingly, the fiber terminals from the BLA largely overlapped with the soma of D1-MSNs but significantly less with D2-MSNs (Fig. 2h). In addition, the projections from the BLA within the DMS displayed a patch-like pattern, largely colocalizing with the mu-opioid receptor (MOR), a marker for the striosome compartments^37^ (Fig. 2i). Next, we confirmed that BLA inputs make functional synapses onto D1-MSNs in the DMS by electrophysiological recordings (Fig. 2j). To measure optically evoked excitatory postsynaptic currents (oEPSCs), we injected AAVs harboring a channelrhodopsin-2 (ChR2) construct (AAV-ChR2-EYFP) into the BLA of D1-tdTomato mice. The stimulus-response curve of oEPSCs recorded in D1-MSNs (tdTomato+) and D2-MSNs (tdTomato-) neurons revealed significantly larger BLA-evoked oEPSCs in D1-MSNs than in D2-MSNs at each light stimulation intensity (Fig. 2k), consistent with the imaging analysis data. Thus, our results indicate that the BLA preferentially innervates D1-MSNs within striosomes in the anterior DMS^38^ and this connectivity might mediate stress-induced compulsive-like behaviors.

### Optogenetic stimulation of BLA-DMS projections increases anxiety and compulsive grooming

**Fig. 3:**
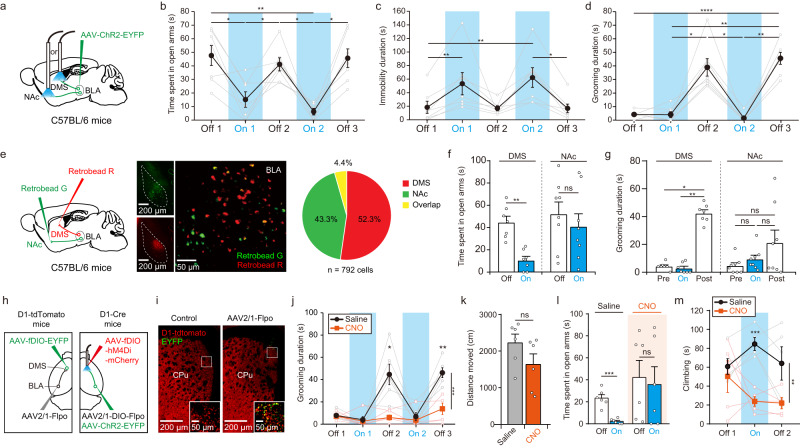
Optogenetic activation of the BLA-DMS circuit induces anxiety-like behavior followed by compulsive grooming. **a** Experimental scheme (ChR2_BLA-DMS_, *n* = 7; ChR2_BLA-NAc_, *n* = 8) used in (**b**–**d**, **f**, **g**). **b**–**d** Behavioral responses in EPM induced by optical stimulation of BLA-DMS. Open arm duration (**b**, one-way repeated measures ANOVA with Greenhouse-Geisser correction and post hoc Tukey’s test, *F*_2.361, 14.17_ = 14.86, *P* = 0.0002), immobility (**c**, two-sided Friedman test and post hoc Dunn’s multiple comparisons test, *H* = 23.09, *P* = 0.0001), and grooming duration (**d**, two-sided Friedman test and post hoc Dunn’s multiple comparisons test (*H* = 23.88, *P* = 8.440e^−05^) showed significant effects of optical stimulation. There was a significant difference between Off1 and Off3 in grooming (paired *t*-test, *t* = 9.690, *df* = 6, *P* = 6.929e^−05^). **e** Scheme (left), representative images (middle), quantification (right, *n* = 4 mice) of retrograde tracing of BLA-DMS and BLA-NAc projections. **f** Anxiety measured in EPM with BLA-DMS *vs* BLA-NAc activation (two-way repeated measures ANOVA with post hoc Sidak’s multiple comparisons test, interaction_light × region_, F_1,13_ = 4.159, *P* = 0.0623; Light effect_,_ F_1,13_ = 16.36, *P* = 0.0014). **g** Grooming behavior induced by BLA-DMS *vs* BLA-NAc activation (two-sided Friedman test with post hoc Dunn’s multiple comparisons test, BLA-DMS, *H* = 11.14, *P* = 0.0012; BLA-NAc, *H* = 1.355, *P* = 0.5444). **h** Schematic of injections in (**i**–**m**). **i** Representative images of anterograde transsynaptic tagging in the DMS. A subset of D1-MSNs expressing EYFP after Flpo-mediated recombination is shown in yellow. This experiment was repeated 3 times with similar results. **j** Inactivation of the target D1-MSNs prevented induction of excessive grooming by BLA-DMS activation (Saline, *n* = 6; CNO, *n* = 6) (Two-way repeated measures ANOVA with post hoc Sidak’s multiple comparisons test, interaction_light × drug_, *F*_4, 40_ = 10.38, *P* = 7.324e^−6^). **k** The total distance traveled in EPM (Saline, *n* = 6; CNO, *n* = 6) (two-sided unpaired t test, *t* = 1.582, *df* = 10, *P* = 0.1447). **l** Open arm duration in EPM (Saline, *n* = 6; CNO, *n* = 6) (two-sided paired *t*-test, saline, *t* = 7.324, *df* = 5, *P* = 0.0007; CNO, *t* = 0.8654, *df* = 5, *P* = 0.4264). **m** Active climbing in FST (Saline, *n* = 5; CNO, *n* = 5) (two-way repeated measures ANOVA with post hoc Sidak’s multiple comparisons test, Drug effect, *F*_1, 8_ = 18.01, *P* = 0.0028).

If PRST modulated defensive behaviors via the BLA-DMS connectivity, it could be possible to induce similar repetitive behaviors by activating the circuit without PRST. To examine the role for the BLA-DMS circuit in stress-related repetitive behavior, we injected AAV-ChR2-EYFP into the BLA and applied optogenetic stimulation to the axon terminals of the infected BLA neurons within the DMS in vivo (Fig. 3a). Activating this circuit in mice induced strong fear- or anxiety-like responses. The mice demonstrated strong freezing responses and significantly lower exploration in an EPM test during optical stimulation compared to pre- and poststimulation conditions (Fig. 3b, c). Interestingly, compulsive grooming behavior was completely inhibited during stimulation but strikingly increased in the poststimulation period (Fig. 3d and Supplementary Movie 1). However, control mice injected with AAV-EYFP did not respond to optical stimulation (Supplementary Fig. 2).

The BLA sends more widespread inputs to the nucleus accumbens (NAc) than to the DMS (Fig. 2a). If the BLA neurons projecting to the DMS have collateral connections to the NAc, then BLA-NAc connections could be co-activated by antidromic activity. However, a double labeling experiment using two different retrograde dyes separately injected into the DMS or NAc revealed that only a few cells in the BLA were doubly labeled, indicating that most DMS-projecting BLA neurons belong to a distinct population from the BLA neurons projecting to the NAc (Fig. 3e). Furthermore, optical stimulation of BLA axons within the NAc caused neither an increase in anxiety nor compulsive grooming (Fig. 3f, g). Therefore, the increase in anxiety and self-grooming observed with optical stimulation is highly likely the consequence of specific BLA-DMS activation.

Since D1-MSNs are the major recipient cells of BLA input within the DMS, we investigated whether D1-MSNs were indeed essential for the manifestation of compulsive behavior. To avoid a general depression of activity, we restricted the expression of an inhibitory DREADD, hM4Di, only to D1-MSNs directly receiving BLA input using a transsynaptic AAV2/1 vector (Fig. 3h). AAV2/1 serotype spreads transsynaptically in anterograde direction in the central nervous system^39^. To confirm the anterograde transneuronal transport of AAV2/1 vector in the BLA-DMS pathway, we first injected AAV2/1-Flpo in the BLA and AAV-fDIO-EYFP in the DMS. We found many EYFP-positive neurons in the DMS of these mice, confirming the transsynaptic transduction of AAV2/1 in BLA-DMS synapses (Fig. 3i). For specific manipulation of D1-MSNs receiving BLA input in the DMS, we injected AAV-ChR2 and AAV2/1-DIO-Flpo into the BLA and AAV-fDIO-hM4Di into the DMS of D1-Cre mice. Whereas CNO administration did not significantly affect locomotor activity or basal level grooming in these mice, it prevented increased poststimulation grooming (Fig. 3j, k). In addition, induction of anxiety and compulsive coping behavior by optogenetic stimulation of the BLA-DMS circuit was also precluded in CNO-treated mice (Fig. 3l, m). This clearly demonstrated that the D1-MSNs receiving input from the BLA play a crucial role in manifesting the stress-induced increase in compulsive behaviors.

### Chronic BLA-DMS activation induces persistent OCD-like phenotypes

**Fig. 4:**
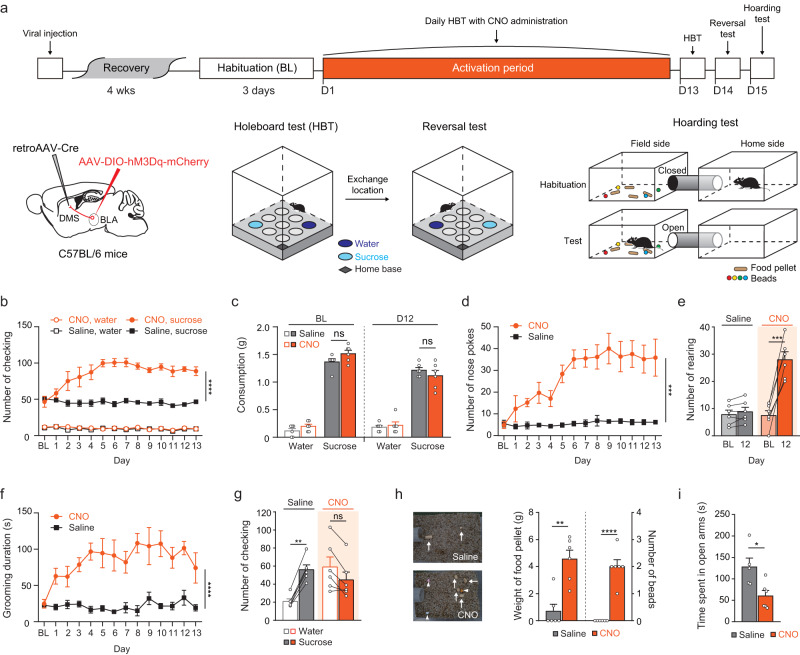
Chronic chemogenetic activation of the BLA-DMS circuit produces various compulsive-like phenotypes. **a** Experimental scheme for chronic BLA-DMS activation and diagrams of behavioral tests. Daily CNO (1 mg/kg) injections (i.p.) were administered for 12 consecutive days after 3 days of habituation period (Saline, *n* = 6; CNO, *n* = 6). **b** Number of repetitive checking (visits) to water or sucrose wells (Two-way repeated measures ANOVA, interaction_day × drug_, *F*_13, 130_ = 6.628, *P* = 1.360e^−09^). **c** Sucrose or water consumptions at baseline (BL) and Day 12 (D12). **d** Number of nose pokes (Two-way repeated measures ANOVA, interaction_day × drug_, *F*_13, 130_ = 6.740, *P* = 9.249e^−10^). **e** Number of rearing (two-sided paired *t*-test, Saline, *t* = 1.732, *df* = 5, *P* = 0.1438; CNO, *t* = 8.244, *df* = 5, *P* = 0.0004). **f** Grooming duration (two-way repeated measures ANOVA, interaction_day × drug_, *F*_13, 130_ = 2.272, *P* = 0.0099). **g** Number of checking occurrences on a reversal test (Two-way repeated measures ANOVA with post hoc Sidak’s multiple comparisons test, interaction_drink × drug_, F_1,10_ = 10.10, *P* = 0.0099). **h** Left, representative images showing the collected food pellets (arrows) and beads (arrow heads). Right, quantification (Food pellets, two-sided Mann–Whitney test, *U* = 1.5, *P* = 0.0065; beads, unpaired *t*-test, *t* = 7.746, *df* = 10, *P* = 1.5603e^−05^). **i** Open arm duration in EPM (Saline, *n* = 5; CNO, *n* = 6) (two-sided unpaired *t*-test, *t* = 2.921, *df* = 9, *P* = 0.017).

Repetitive behaviors observed in psychiatric disorders are often persistent. To examine whether chronic hyperactivity in the BLA-DMS circuit leads to perseverative behaviors, we next investigated the effect of prolonged activation of the circuit. For selective, repeated activation of the BLA-DMS circuit, we injected a retrograde AAV harboring Cre (retroAAV -Cre) into the DMS and an AAV-DIO-hM3Dq into the BLA. After four weeks of recovery, the animals received daily intraperitoneal CNO injections for 12 days (activation period; Fig. 4a). To examine repetitive checking and exploratory behaviors, we subjected animals to a modified holeboard test in which we placed a water-containing well (WW) in a corner and a 10% sucrose solution-containing well (SW) in the opposite corner of a holeboard^40^. After three days of pre-activation habituation, all mice visited the SW significantly more than the WW and drank more sucrose solution than water. While saline injections during the activation period did not affect the number of visits to both the WW and the SW, CNO injections caused a dramatic increase in the number of visits to the SW, but not to the WW (Fig. 4b and Supplementary Movie 2). However, the total consumption of sucrose solution did not differ between CNO-treated mice and saline-treated mice, indicating that the higher number of visits to the SW in CNO-treated mice reflected more frequent checking rather than increased consumption (Fig. 4c). CNO-treated mice also showed a significant increase in the number of exploratory nose pokes into empty holes as well as in rearing (Fig. 4d, e). Furthermore, compulsive grooming was also significantly increased (Fig. 4f). These increased compulsive-like behaviors shared similar kinetics, reaching plateau approximately after one week of daily CNO administrations. Impaired cognitive flexibility has been proposed to be associated with compulsions, although there are variable findings in clinical studies^41^. We, therefore, sought to test the behavioral adaptability of our mice by a reversal test. Interestingly, CNO-treated mice did not show a significant difference in the number of visits to WW (formerly having a sucrose solution) and SW while control mice visited SW significantly more than WW. There was a statistically significant interaction between the effects of treatment (Saline/CNO) and location (Water/Sucrose) on checking (Fig. 4g). This indicates that CNO-treated mice continued to compulsively check the same position despite a change in reward value. We additionally examined whether hoarding behavior could be induced by chronic activation of the BLA-DMS circuit. We placed animals into one compartment of the testing chamber (home side), which was connected by a corridor to another compartment (field side) that contained food pellets and plastic beads. Animals were habituated in the home side with the corridor closed during day and allowed to explore the field side at night. Surprisingly, the CNO-treated mice not only carried significantly more food pellets than control mice, but also collected a few beads overnight. In contrast, saline-treated control animals did not carry any non-food items (Fig. 4h and Supplementary Movie 3). Furthermore, these compulsive-like behaviors were accompanied by heightened anxiety, as measured in EPM test, indicating the interrelationship between anxiety and compulsions (Fig. 4i). To address the concerns about nonspecific effects due to a metabolized product of CNO, clozapine^42^, we used a minimal effective dose of CNO, which has been shown not to produce detectable clozapine in plasma^43^. Additionally, we chronically administered the CNO dose used in this study to mice in the absence of hM3Dq expression and found no significant change in behavior (Supplementary Fig. 3). We also have used another chemogenetic actuator, deschloroclozapine (DCZ), for chronic activation of BLA-DMS circuit and obtained very similar results to when using CNO (Supplementary Fig. 4). DCZ was shown to be highly selective for hM3Dq without off-target side effects unlike clozapine^44^. These results indicate that the compulsive behaviors observed in our study were caused by BLA-DMS circuit activation, not by a nonspecific effect of reverse-metabolized clozapine.

**Fig. 5:**
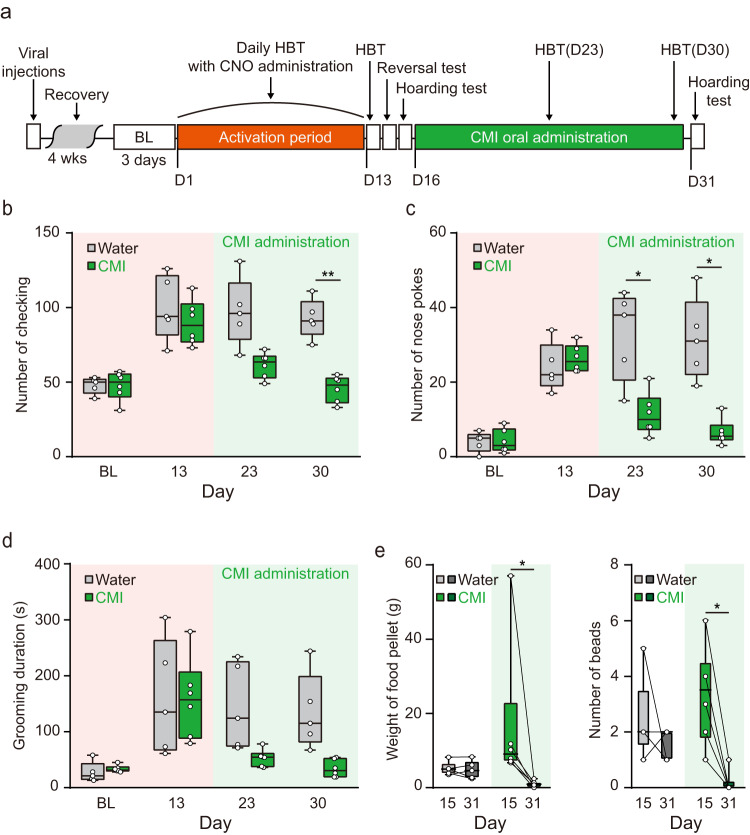
Clomipramine (CMI) can relieve the compulsive-like symptoms induced by chronic activation of the BLA-DMS circuit. **a** Experimental scheme. After the activation period, animals were provided with CMI-containing water for 14 days. CMI concentration was adjusted to an average 20 mg/kg CMI intake per day (Water, *n* = 5; CMI, *n* = 6). **b**–**d** Behavioral responses of mice model for compulsivity to CMI administration (two-way repeated measures ANOVA with post hoc Sidak’s multiple comparisons test, interaction_day × drug_ (checking), *F*_3, 27_ = 8.784, *P* = 0.0003) (**b**), interaction_day × drug_ (number of nose pokes), *F*_3, 27_ = 12.83, *P* = 2.131e^−05^ (**c**), interaction_day × drug_ (grooming duration), *F*_3, 27_ = 4.769, *P* = 0.0086 (**d**)). Box-and-whisker plots display the median (line), 25th and 75th percentiles (box edges), minimum and maximum (whiskers). **e** Effects of CMI on hoarding behavior (Two-sided Wilcoxon matched-pairs signed rank test, food pellets, Median = −8.05, *P* = 0.0313; beads, Median = −3.5, *P* = 0.0313). Box-and-whisker plots display the median (line), 25th and 75th percentiles (box edges), minimum and maximum (whiskers).

Since these mice displayed many compulsive-like behaviors, we next questioned whether these mice would respond to a known therapeutic treatment used for human compulsive disorders. Serotonin reuptake inhibitors (SRIs) are often a first-line treatment for patients with OCD^45^. Therefore, we tested whether the administration of an SRI, clomipramine (CMI), could alleviate the compulsive-like behaviors of these mice. After the activation period, CNO-administered mice were divided into a control group provided with plain water and a CMI group which received drinking water containing CMI (20 mg/kg body weight per day). In the control group, compulsive-like behaviors, including hoarding, persisted for up to two weeks after the last CNO administration while those behaviors were dramatically alleviated in CMI-treated animals (Fig. 5).

### Mitogen-activated protein kinase (MAPK) pathway activation in the BLA-DMS circuit mediates the compulsive-like phenotypes

**Fig. 6:**
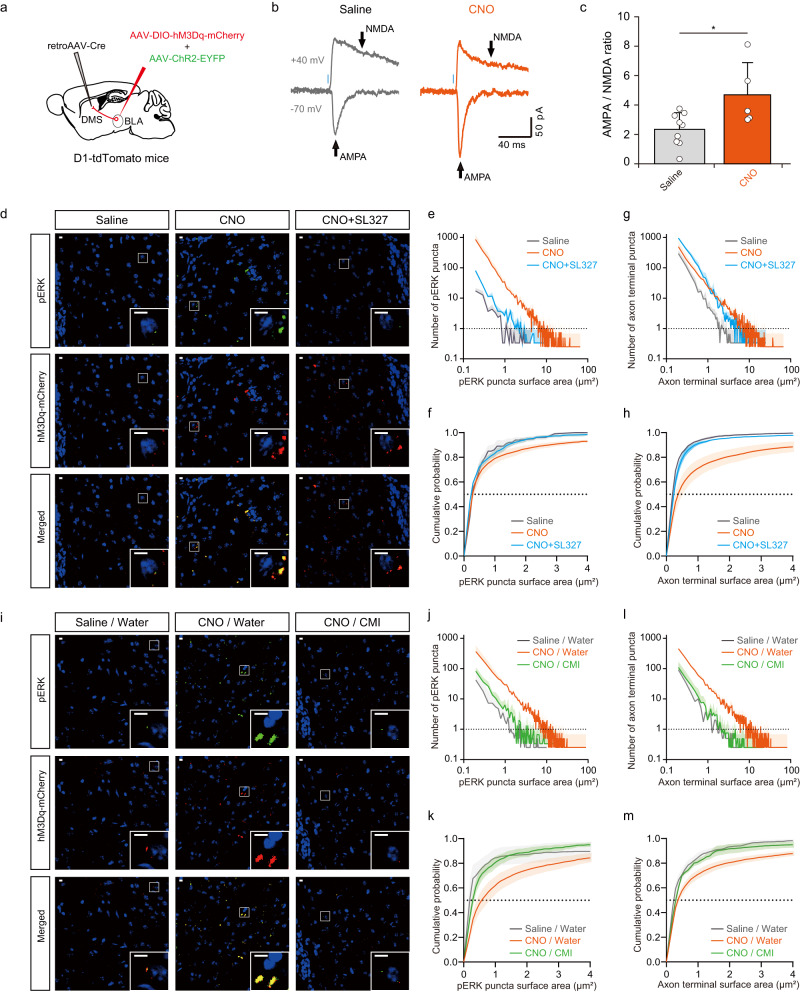
Chronic chemogenetic activation of the BLA-DMS circuit increases ERK phosphorylation levels and synaptic plasticity in the DMS. **a** Scheme of virus injection for optical stimulation-evoked excitatory postsynaptic current (oEPSC) measurement. **b** Representative oEPSC traces measured in D1-MSNs (tdTomato+). Blue rectangles indicate times of light stimulation (470 nm, 1 ms) and arrows indicate sampling time of AMPA receptor- and NMDA receptor-mediated currents. **c** AMPA/NMDA ratio in control (*n* = 9 cells from 3 mice) and CNO-treated mice (*n* = 5 cells from 4 mice) (two-sided Student’s *t*-test, *t* = 2.6989, *df* = 12, *P* = 0.0193). **d**−**h** Immunohistochemistry using pERK antibody performed on D13−D16 (Saline, *n* = 3 mice; CNO, *n* = 4 mice; CNO + SL327, *n* = 3 mice). Representative images are shown in (**d**) where enlarged images showing the colocalization of pERK with BLA axons (hM3Dq-mCherry) are displayed in insets. Scale bars indicate 50 µm. Quantified data of pERK puncta and BLA axons are shown in (**e**) and (**g**), respectively (two-sided Friedman test with post hoc Dunn’s multiple comparisons test, pERK: Saline *vs* CNO, *P* = 1.0e^−15^; CNO *vs* CNO + SL327, *P* = 1.0e^−15^; Saline *vs* CNO + SL327, *P* = 0.4911; Axon: Saline *vs* CNO: *P* = 1.0e^−15^; CNO *vs* CNO + SL327, *P* = 1.0e^−15^; Saline *vs* CNO + SL327, *P* = 7.286e^−08^). The pERK puncta and BLA axons data shown in (**e**) and (**g**) are represented in a cumulative probability plot in (**f**) and (**h**), respectively (two-sided Kolmogorov−Smirnov test, pERK: Saline *vs* CNO, *P* = 0.0259; CNO *vs* CNO + SL327, *P* = 0.002; Saline *vs* CNO + SL327, *P* = 0.8186; Axons: Saline *vs* CNO, *P* = 1.621e^−12^; CNO *vs* CNO + SL327, *P* = 1.0e^−15^; Saline *vs* CNO + SL327, *P* = 0.0028). **i**−**m** Immunohistochemistry using a pERK antibody performed after CMI administration (D31-D32) (Axon terminals analysis: Saline/Water, *n* = 4 mice; CNO/Water, *n* = 4 mice; CNO/CMI, *n* = 4 mice, pERK analysis: Saline/Water, *n* = 4 mice; CNO/Water, *n* = 4 mice; CNO/CMI, *n* = 3 mice). Representative images are shown in (**i**) in which enlarged images showing the colocalization of pERK signals with BLA axons (hM3Dq-mCherry) are displayed in insets. Scale bars indicate 50 µm. Quantified data of pERK puncta and BLA axons are shown in (**j**) and (**l**), respectively (two-sided Friedman test with post hoc Dunn’s multiple comparisons test, pERK: Saline/Water *vs* CNO/Water, *P* = 1.0e^−15^; CNO/Water *vs* CNO/CMI, *P* = 1.0e^−15^; Saline/Water *vs* CNO/CMI, *P* = 0.4550; Axon: Saline/Water *vs* CNO/Water, *P* = 1.0e^−15^; CNO/Water *vs* CNO/CMI, *P* = 1.0e^−15^; Saline/Water *vs* CNO/CMI, *P* = 0.0290). The pERK puncta and BLA axons data shown in (**j**) and (**l**) are represented in a cumulative probability plot in (**k**) and (**m**), respectively (two-sided Kolmogorov−Smirnov test, pERK: Saline/Water *vs* CNO/Water, *P* = 8.184e^−09^; CNO/Water *vs* CNO/CMI, *P* = 9.225e^−08^; Saline/Water *vs* CNO/CMI, *P* = 0.0222; Axon: Saline/Water *vs* CNO/Water, *P* = 3.72e^−09^; CNO/Water *vs* CNO/CMI, *P* = 1.564e^−08^; Saline/Water *vs* CNO/CMI, *P* = 0.0062).

Unlike the compulsive grooming induced by acute optogenetic stimulation, behaviors resulting from repetitive stimulation of the BLA-DMS circuit lasted for an extended period of time after the activation period ended. This suggested that accompanying molecular and/or physiological changes might underlie the persistent compulsive-like behaviors in these mice. Thus, we first searched for molecular changes associated with behavioral changes in mice showing compulsive-like phenotypes. Persistent molecular and physiological changes that are induced by repeated synaptic stimulations are the essential feature of synaptic plasticity^46^. To investigate the synaptic changes induced by chronic BLA-DMS circuit activation, we injected AAV-ChR2-EYFP and AAV-DIO-hM3Dq-mCherry into the BLA and retroAAV-Cre into the DMS (Fig. 6a and Supplementary Fig. 5), and measured AMPA and NMDA receptor-mediated BLA-evoked oEPSCs in D1 neurons. We found that the D1-MSNs of mice that underwent prolonged activation of the BLA-DMS circuit displayed a significantly greater AMPA/NMDA ratio of oEPSCs than those of control mice (Fig. 6b, c). NMDA receptor-dependent long-term potentiation (LTP) has been shown to occur by AMPA receptor redistribution, which changes the AMPA/NMDA ratio of excitatory currents^47,48^. Therefore, we hypothesized that structural or molecular events associated with synaptic plasticity might have occurred at the BLA-DMS synapses in these mice. A molecule crucially involved in triggering LTP is the extracellular signal-regulated kinase (ERK), a subfamily of MAPK^49,50^. To examine whether ERKs played a role in the manifestation of compulsive-like behaviors in these mice, we first analyzed the level of phosphorylated ERK (pERK), an active form of ERK, in the BLA-DMS circuit at the end of 12-day activation period. The pERK level dramatically increased in the DMS and in the BLA of CNO-treated mice (Fig. 6d, e, f and Supplementary Fig. 6). Interestingly, CNO-treated mice displayed significantly more, larger signal puncta representing the axons of the BLA-DMS projections than control mice, suggesting that some modifications to the presynaptic terminals might have occurred at the BLA-DMS synapses (Fig. 6d, g, h). We next examined whether the alleviation of compulsive-like behavior caused by two weeks of CMI administration (Fig. 5) could be correlated with changes in pERK expression. We found that the levels of both pERK and the axonal arbors from the BLA neurons in CMI-treated mice were indistinguishable from those in saline-treated mice. However, CNO-treated mice that drank regular water for the 2-week period maintained the increased pERK level and axonal arbors (Fig. 6i–m). This change in pERK expression was also detected by immunobloting (Supplementary Fig. 7). Next, we tested whether increased neural activity in the BLA-DMS circuit per se could activate the MAPK pathway in the DMS. To this end, we expressed ChR2 in the BLA and applied unilateral optical stimulation to the axons in the DMS. This optogenetic stimulation significantly increased colocalization of axon terminals and pERK signals, indicating that increased activity of BLA neurons can result in MAPK pathway activation (Supplementary Fig. 8).

**Fig. 7:**
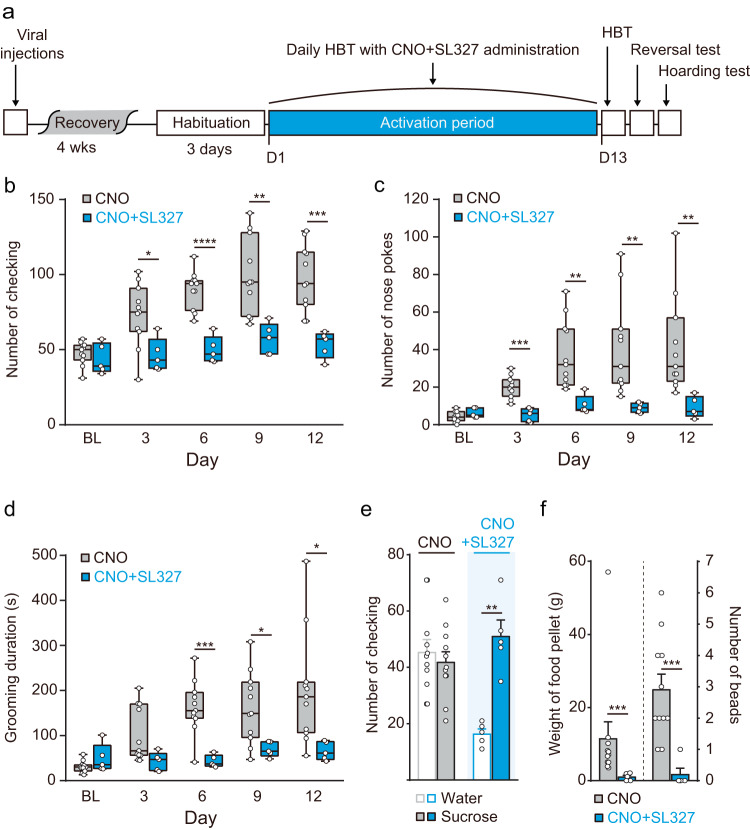
Blocking the MAPK/ERK pathway during the activation period prevents compulsive-like behaviors in mice with chronic BLA-DMS activation. **a** Experimental scheme. SL327 along with CNO was administered for 12 consecutive days after the habituation period. SL327 or saline was administered 15 min before CNO injections. (CNO (+Saline), *n* = 11; CNO + SL327, *n* = 5). **b**–**d** Behavioral outcomes of co-administration of SL327 with CNO during the activation period (two-way repeated measures ANOVA with post hoc Sidak’s multiple comparisons test, interaction_day × drug_ (checking), *F*_4, 56_ = 6.411, *P* = 0.0003 (**b**), interaction_day × drug_ (nose pokes), *F*_4, 56_ = 5.427, *P* = 0.0009 (**c**), interaction_day × drug_ (grooming duration) *F*_4, 56_ = 4.283, *P* = 0.0043 (**d**)). Box-and-whisker plots display the median (line), 25th and 75th percentiles (box edges), minimum and maximum (whiskers). **e** Effect of SL327 on reversal test (two-sided paired *t*-test, CNO, *t* = 1.455, *df* = 10, *P* = 0.1762; SL327 + CNO, *t* = 6.898, *df* = 4, *P* = 0.0023). **f** The effect of SL327 on hoarding behavior (two-sided Mann−Whitney test, Food pellet, *U* = 0, *P* = 0.0005; Beads, *U* = 1, *P* = 0.0009).

Given that MAPK pathway activation within the BLA-DMS circuit was highly correlated with the development of compulsive-like behaviors, we hypothesized that inhibiting the MAPK pathway during the repeated stimulation period would preclude the emergence of compulsive-like behaviors. Thus, we administered SL327, a brain-permeable MAPK/ERK kinase inhibitor, during the activation period along with CNO to suppress ERK activation (Fig. 7a). SL327 administration significantly prevented the activation of ERK and the concomitant enlargement of axonal arbors in the DMS (Fig. 6d–h) and effectively suppressed the development of compulsive-like behaviors (Fig. 7b–f), demonstrating that MAPK pathway activation is essential for manifesting and/or maintaining compulsive-like symptoms in this mouse model.

## Discussion

Our study demonstrates the functional importance of the BLA-DMS circuit in the manifestation of compulsivity-like behaviors in mice. We showed that highly repetitive behaviors could be induced by specific activation of D1-MSNs within the DMS with prior exposure to PRST, which activated neurons in the anterior BLA. PRST is an ethologically-relevant stress for animals and PRST-induced defenses can be useful models of human emotional disorders^51^. PRST has been shown to induce general decreases in locomotor activity and active avoidance behavior. Although how these apparently conflicting behaviors are regulated is not clearly known, active defensive behavior under threat appear to be dependent on the amygdala and the striatum^52^. Furthermore, active avoidance response was shown to engage BLA while inhibiting freezing response by preventing lateral amygdala from activating the freezing circuits of the CEA^53,54^. Our data might imply that compulsive behaviors can result from maladaptive activation of the circuit subserving defensive avoidance behavior.

We then demonstrated that there are direct and functional connections between BLA neurons and D1-MSNs in the DMS. We found that the specific optogenetic activation of this circuit in vivo induced strong anxiety-like behaviors. The BLA contains a critical node of network for valence encoding and assigning valence to sensory stimuli^55,56^. Our data demonstrate that the negative valence neurons in the anterior BLA preferentially projecting to the DMS induce anxiety-like behavior associated with compulsive-like behaviors. As for the role of BLA-NAc connections, there have been mixed findings^17,20,34,35,57,58^, likely due to the genetic and anatomical heterogeneity of valence-encoding neurons in this circuit. Although many DMS-projecting BLA neurons were negative-valence neurons (activated by PRST), whether they also include positive valence neurons remains unclear. Nonetheless, the BLA-DMS circuit seems to play a distinct functional role from the BLA-NAc circuit in that it induces an anxiety-compulsive behavior coupling.

The striatum comprises two distinct compartments, striosomes and matrix. The striosome compartment spans the striatum with three-dimensional labyrinthine structures that interdigitate a larger surrounding matrix compartment^59^. The striosome and the matrix have been defined by different patterns of histochemical staining for marker genes such as acetylcholinesterase, MOR, substance P, enkephalin. While both D1-MSNs and D2-MSNs reside in either the strosome or matrix compartment, it appears that there are more D1-MSNs than D2-MSNs in striosomes, at least in the anteromedial part of the striatum^38,60^. This part of the striosomal system has been related to the limbic system since it is innervated by many regions of the limbic system^61^, including the anterior cingulate cortex, the orbitofrontal cortex, the bed nucleus of the stria terminalis, and the BLA^62^. Thus, it is feasible that the PRST-responsive neurons in the anterior BLA innervate the striosomes in the anteromedial striatum and this circuit contributes to behavioral control as a part of negative valence systems that are responsible for responses to aversive context such as stress, fear, and anxiety. Recently, a subset of striosomal D1-MSNs that express the *Teashirt family zinc finger 1* (*Tshz1*) has been shown to be essential for avoidance and to represent the motivation to avoidance^63^. It is feasible that the PRST-responsive neurons in the BLA project to the *Tshz1*-expressing striosomal cells in the DMS to play a role in the manifestation of compulsions.

While striosomes and striosome-connected circuits are involved in decision-making under cost-benefit conflict and value-based learning in normal conditions^64–66^, possibly by coordinated interplay between neurons receiving positive valence information and neurons accepting negative valence information, dysregulation of the circuit may produce compulsions. In fact, heightened activity of a small area within the striatum caused either by stimulant drug or microstimulation has been demonstrated to be correlated or to induce persistent and repetitive behaviors^67,68^. These findings are comparable to ours where compulsive grooming was induced by the activation of the BLA-DMS circuit, which subsequently activated D1-MSNs, possibly within striosomes. However, how compulsive grooming was induced in the poststimulation period of optogenetic stimulation experiments is unclear. Substantia nigra pars compacta (SNc) neurons respond to aversive stimuli by inhibitory pause and subsequent rebound activation^69^. Striosomes selectively innervate the ventral population of dopaminergic neurons in the SNc forming an unusual bouquet-like arborizations and cause SNc neurons to pause and rebound^70,71^. In fact, we found that DMS neurons that receive BLA inputs project to the SNc and have an arborization pattern resembling the striosome-dendron bouquet structure (Supplementary Fig. 9). It is therefore intriguing to postulate that aversive stimuli can induce SNc neurons to pause and rebound via the BLA-DMS (striosome)-SNc connection and that the rebound activation of SNc neurons can initiate compulsive grooming. Future research should determine whether phasic dopamine release from ventral SNc neurons is involved in the pathophysiology of compulsions.

Our chronic BLA-DMS activation model has produced various compulsive-like behaviors such as checking, grooming, and stereotypy. In addition, our model displays a hoarding behavior that might be related to human hoarding disorder in that they collect apparently worthless items. Interestingly, these compulsions increase over time with the same daily CNO dose. Furthermore, the increased level of compulsive-like behaviors was maintained without further activation of the circuit for ≥2 weeks. This gradual but lasting increase in compulsions resembles the behavioral sensitization caused by addictive stimulant drugs, suggesting the possible induction of some form of synaptic plasticity by repetitive activation of the circuit^72^. Our data indeed support this hypothesis. Changes in AMPA/NMDA ratio are often used to indicate synaptic plasticity at excitatory synapses^73^. The D1-MSNs receiving BLA inputs in our model mice displayed a significant increase in AMPA/NMDA ratio after chronic CNO treatment, which strongly supports a form of long-term plasticity, very likely a strengthening, has been induced at BLA-DMS synapses in our animal model for persistent compulsion. Additionally, the level of pERK, a molecular marker that is closely associated with synaptic plasticity, was dramatically elevated at BLA-DMS synapses in our mice. The Ras-ERK pathway has been strongly implicated in synaptic plasticity both in the hippocampus and the striatum^74,75^. The ERK pathway has also been implicated in the pathogenesis of OCD in a study using mice deficient for SPRED2, a potent inhibitor of Ras/ERK-MAPK signaling^25^. We demonstrated that pERK upregulation as well as compulsive behaviors were maintained for several weeks after chronic stimulation and that the alleviation of compulsive behaviors by CMI administration was accompanied by decrease in pERK signaling in the striatum, implying a functional involvement of the MAPK pathway in the manifestation and persistence of compulsive behaviors. Furthermore, co-administration of an inhibitor of the MAPK pathway, SL327, along with CNO during the activation period precluded not only ERK activation but also the development of compulsive-like behaviors. Our data strongly suggest that synaptic enhancement along with the upregulation of Ras/ERK pathway underlie the development of persistent compulsive-like behaviors.

Overall, our study demonstrates that the BLA-DMS circuit plays a critical role in inducing compulsive-like behaviors that manifest with anxiety. Especially our chronic activation model shows several different compulsive-like behaviors. In addition, the compulsive-like behaviors could be clearly alleviated by administering a tricyclic antidepressant, CMI, which is used to treat OCD. Therefore, our chronic model for compulsivity can be useful for studying the neural mechanisms of OCD and for developing novel therapeutic approaches for OCD. Many clinical and preclinical studies implied the functional importance of CSTC circuit in the pathophysiology of OCD. Particularly, the functional and structural orbitofrontal cortex (OFC) alterations have been shown to be associated with OCD^76^. Furthermore, studies using optogenetic stimulation of the OFC-striatum circuits have shown that compulsive grooming behavior could be altered in mice with the OFC-striatum circuit manipulation^28,77^. The OFC is implicated in processing information related to positive value of predicted outcome and thereby guiding or adapting behavioral choices^78^. Dysregulation of the OFC-striatum circuit therefore might lead to compulsive behavior by exaggerated outcome value prediction of an action and reinforcement. In contrast, negative affective states also need to be considered in the action selection process, sometimes in urgent situations such as in the presence of predators. Our data suggest that the BLA-DMS circuit might serve as an emergency pathway for behavioral adaptation in threatening situations and the persistent maladaptation of this circuit might lead to compulsive-like behaviors.

## Methods

### Animals

All experiments were conducted in compliance with the National Institutes of Health Guide for the Care and Use of Laboratory Animals and all experimental procedures in this study were approved by the Korea University Institutional Animal Care and Use Committees. We used 8 to 20-week-old mice and over 12 weeks old rats in our study. For D1- or D2-MSN chemogenetic activation experiments, we used both male and female mice given the lack of significant differences in behavior between male and female mice in these experiments. Only male mice were used in all other experiments to avoid possible complications due to gender differences in OCD^79^.

Transgenic mice used in this study [Drd1a-tdTomato (B6.Cg-Tg(Drd1a-tdTomato)6Calak/J, termed D1-tdTomato in our study), Drd1a-EGFP (Tg(Drd1-EGFP) × 60Gsat/Mmmh, termed D1-EGFP in our study), Drd2-EGFP (Tg(Drd2-EGFP)S118Gsat/Mmnc, termed D2-EGFP in our study), and Drd1a-CRE (B6.FVB(Cg)-Tg(Drd1-cre)EY217Gsat/Mmucd, termed D1-Cre in our study), Drd2a-CRE (B6.FVB(Cg)–Tg(Drd2-cre) ER44Gsat/Mmucd, termed D2-Cre in our study)] were purchased from the Jackson Laboratory and Mutant Mouse Resource and Research Center. C57BL/N mice and Crl:CD(SD) rats were purchased from Orient Bio.

Animals were maintained under controlled temperature (23 °C) and humidity conditions (50%) with a 12-h light-dark cycle (light on at 7 AM). Food and water were available *ad libitum* except for water deprivation (overnight) experiment for c-Fos measurement.

### Drug treatment

Clozapine N-oxide (CNO; Tocris, Bristol, United Kingdom) and Deschloroclozapine dihydrochloride (DCZ; Hellobio; Bristol, United Kingdom) were freshly prepared in saline and injected intraperitoneally (i.p.) 30 min prior to behavioral tests (CNO: 1 or 10 mg/kg, DCZ: 0.2 mg/kg). SL327 (Tocris, Bristol, United Kingdom), was administered i.p. (30 mg/kg) 15 min before CNO injection. Clomipramine (CMI; Sigma, St. Louis, USA) was added to drinking water at a concentration of 40 mg/L to reach an average dose of 20 mg/kg per day.

### Virus preparation

The pAAV-EF1a-hChR2(H134R)-EYFP, pAAV-EF1a-EYFP, pAAV-hSyn-DIO-hM3D(Gq)-mCherry, pAAV-CAG-tdTomato, pAAV:ITR-U6-sgRNA(backbone)-pCBh-Cre-WPRE-hGHpA-ITR(AAV-Cre), pAAV-EF1a-DIO-Flpo, pAAV-EF1a-Flpo, pAAV-EF1a-fDIO-hChR2(H134R)-EYFP, pAAV-hSyn-fDIO-hM4D(Gi)-mCherry-WPREpA plasmid vectors were used to construct viruses using the AAV Helper-Free System (Cat#240071, Agilent Technologies, Santa Clara, CA, USA). For AAV preparation, AAV-293 cells were cultured until reaching 70–80% confluence and transfected with three plasmids: the pAAV expression plasmid, pAAV-RC (DJ) or pAAV2/1, and pHelper (or rAAV2-retro helper for retroAAV-Cre packaging). The cell culture medium was changed 6 h after transfection, and cells were incubated for an additional 72 h. Cells and growth medium then were collected and subjected to four rounds of freeze/thaw cycles. Then, the cell lysate was centrifuged at 10,000 × *g* for 10 min and the supernatant mixed with one part 40% (w/v) polyethylene glycol and kept at 4 °C for 48 h. The precipitated viral particles were separated at 2000 × *g* for 30 min at 4 °C and the pellet was resuspended in ice-cold PBS. Resuspended viruses were further concentrated by centrifugation at 29,000 × *g* for 2 h at 4 °C. Finally, the pellet was resuspended in ice-cold PBS (1/100 of the starting volume) and stored at −80 °C.

The viral titer was measured by determining the viral DNA concentration. The virus stock was mixed with lysis buffer overnight at 4 °C. Following phenol extraction and separation at 13,200 rpm for 10 min, the supernatant was incubated at −80 °C for 1 h in two parts of 100% ethanol, 1/10 parts of sodium acetate, and 20 mg/mL glycogen. After incubation, DNA was precipitated by centrifugation at 12,000 rpm for 10 min at 0 °C and washed with 70% ethanol. After drying, the DNA was resuspended in distilled water and its concentration was determined by measuring its absorbance using a Nanodrop® spectrophotometer.

### Stereotaxic injection

Stereotaxic injection was performed on 8- to 10-week-old mice. Mice were anesthetized with a ketamine (100 mg/kg; Yuhan corporation, Seoul, Korea) and xylazine (10 mg/kg; Bayer Korea, Seoul, Korea) mixture (i.p.). Mice were placed on a stereotaxic stage (David Kopf instrument, Tujunga, CA, USA), and small holes were drilled bilaterally on the target region. We used a nanoinjector (Nanoliter 2000, World Precision Instrument, Sarasota, FL, USA) for virus or retrograde tracer ({Cholera Toxin Subunit B (Recombinant), Alexa Fluor™ 594 Conjugate, Invitrogen, Carlsbad, CA, USA}, {Retrobeads™ IX [Red or Green], Lumafluor}) injections. The stereotaxic coordinates used for DMS injection were: anteroposterior (AP) + 1.1 mm, mediolateral (ML) ± 1.1 mm, dorsoventral (DV) −3.0 mm; for BLA: AP −1.5 mm, ML ±3.1 mm, DV −4.9 mm; for NAc: AP +1.1 mm, ML ± 1.3 mm, and DV −4.7 mm. The injection pipette was maintained in the brain for an additional 5 min after finishing with the micro-infusion to allow the virus to fully diffuse. All animals were allowed to recover after surgery for ≥3 weeks.

### Optogenetic stimulation

For optogenetic experiments, a patch cable (200 μm diameter with a 0.22 numerical aperture, Doric) was connected to a surgically implanted metal ferrule (MM-FER2007-304-2300, Precision Fiber Products). Optical stimulation was performed using a pulse generator (BNC model 575) connected to a blue laser source (473 nm; MBL-FN-473-150mW, CNI Optoelectronics Tech Co.). Blue light (5 ms duration width, 473 nm) was delivered at a frequency of 20 Hz. Before every optical stimulation experiment, the light power (8–10 mW) was tested at the tip of patch cable.

For chronic optical stimulation of the BLA-DMS circuit, unilateral stereotaxic injection of virus was conducted, and optic fiber was implanted ipsilaterally. The BLA coordinates for virus injection and DMS coordinates for fiber implantation were the same as above. At 3–4 weeks after surgery, grooming duration was measured by daily optical stimulation in an open field (30 cm × 30 cm × 40 cm, white acrylic chamber). Chronic optical stimulation was conducted for five consecutive days. A day before chronic optical stimulation, mice were habituated to an open field chamber with patch cable connection but without optical stimulation. Prior to each optical stimulation, all mice were habituated under dim light for 15 min, followed by habituation with a patch cable connection on ferrule for 15 min in their home cage. Mice received optical stimulation for 15 min divided into five equal phases (laser off 3 min, on 3 min, off 3 min, on 3 min, and off 3 min). The laser settings for optical stimulation were the same as above. All mice were perfused within 15 min after optical stimulation.

### Behavioral tests

All behavioral tests were conducted in a soundproof chamber under dim light conditions (20 lux) with video recordings. Animal behaviors were analyzed either with [EthoVision XT 11.5 software (Noldus, Wageningen, Netherlands)] (EPM) or manually (Holeboard test, FST, Hoarding). In holeboard test, checking behavior was analyzed for 1 h and grooming, nose pokes were analyzed for 15 min. All manually evaluated behavioral assessments were performed in a blind fashion by trained researchers.

#### Predator stress exposure

Mice were placed in a rectangular plastic container (52 cm × 68 cm × 44 cm) containing a cage with two rats [Crl:CD (SD), 450–600 g]. A nontransparent divider was placed in the middle of the container to prevent visual contact. Mice were allowed to be exposed to the odor and sound made by the rats overnight for 16–18 h.

#### Elevated plus maze (EPM) test

The EPM consisted of open (70 cm × 7 cm × 0.5 cm) and closed arms (70 cm × 7 cm × 17 cm), raised 57 cm above the floor. Mice were first habituated for 15 min and an additional 15-min habituation was allowed after connecting the patch cables to the pre-implanted ferrule in optogenetic experiments. For chemogenetic experiments, a CNO i.p. injection was administered 30 min before the test. Mice used for optogenetic experiments were subjected to a 15-min session, divided into five 3-min stimulation off- or on-phases. For EPM tests without optical stimulation, mice were subjected to a 5-min session.

#### Forced swim test (FST)

For the FST, the animals were placed in an acrylic cylinder (25 cm height × 10 cm diameter) filled with 1 L of distilled water (23 °C ± 1 °C). The mice were allowed to swim for 6 min. For experiments using chemogenetics, CNO i.p. injection was administered 30 min before the experiments. For experiments using optical stimulations, mice were subjected to a 6-min session, divided into three 2-min stimulation off- or on-phases (off-on-off). An upward movement of the forepaws against the side of the cylinder was considered climbing.

#### Holeboard test

A 30 cm square white acrylic platform with nine holes (3 cm in diameter) was placed at 7.5 cm above the floor in a transparent acrylic chamber (30 cm × 30 cm × 40 cm). We assigned one corner as a home base by placing nesting material. A water-containing well was placed to the next corner to home base and a 10% sucrose-containing well was placed in the opposite corner to the water well. A mouse was placed in the home base and allowed to explore the chamber without any restriction for 1 h. For every session, the sucrose and water consumption was measured. We started chronic CNO injections when mice had learned the position of the sucrose well. The learning criterion was sucrose consumption/(sucrose + water consumption) ≥ 0.8.

#### Hoarding test

A hoarding test chamber was constructed using two cages connected by a passage tunnel (PVC cylinder with 5 cm diameter and 9.5 cm in length). One was designated as a home side and the other as field side. The home side was provided with food and water during habituation. Mice were habituated in the home side for two days with a blocked passage tunnel. For the hoarding test, food was removed from the home side and food pellets and small plastic beads were placed in the field side. Mice were allowed to explore the field side by opening the passage overnight. The next morning, the amount of food pellets and plastic beads (of different shapes, 0.5 cm in diameter and 0.2–0.9 g in weight) brought to the home side was measured.

### Immunohistochemistry

A mixture of ketamine (100 mg/kg) and xylazine (10 mg/kg) was used for anesthetizing animals and transcardiac perfusion was conducted with ice-cold phosphate buffered saline (PBS) and 4% ice-cold paraformaldehyde (PFA) in PBS (Sigma, St. Louis, MO). Brains were additionally fixated in 4% PFA for 4 h at 4 °C and moved to 30% sucrose in PBS for ≥48 h at 4 °C. For cryosections, we embedded the mouse brain using an O.C.T compound (Tissue-Tek, Sakura Finetek, Netherland) in 0.1 M PBS and sectioned the brain into 40-μm-thick slices, which were maintained at −20 °C in 50% glycerol and a 1 M PBS mixture. Slices were washed three times with 0.1 M PBS for 5 min, followed by three 5-min washes with PBST (0.3% Triton X-100 was used for pERK and TH and 0.5% Triton X-100 was used for c-Fos and the mu-opioid receptor, MOR). After permeabilization, slices were incubated in blocking solution (bovine serum albumin (BSA) in PBST: 3% BSA for c-Fos, pERK and MOR; 5% normal goat serum supplemented with 0.1% BSA for TH). The incubation with primary antibodies was performed overnight at 4 °C with antibodies against c-Fos (1:2000, abcam, Cambridge, UK), MOR (1:400, Abcam, Cambridge, UK), TH (1:500, Millipore, Burlington, MA, USA), and Phospho-p44/42 MAPK (1:400, Cell Signaling, Danvers, USA). After overnight incubation, the slices were washed three times with PBST for 5 min and incubated with the secondary antibodies Alexa Fluor 488 donkey anti-rabbit (1:400, Invitrogen, Carlsbad, CA, USA) for c-Fos, TH, and pERK or Alexa Fluor 594 donkey anti-rabbit (1:400, Invitrogen, Carlsbad, CA, USA) for MOR for 1 h at room temperature. Then, the slices were washed three times in PBST for 5 min, mounted on slides, and protected with antifade mounting medium (VECTASHIELD, Vector Laboratories, Burlingame, CA, USA). pERK and MOR slices were additionally stained with Hoechst 33342 (1:1000, Invitrogen, Carlsbad, CA, USA) for 10 min in 0.1 M PBS.

### Imaging and image analysis

All images were acquired on a Zeiss LSM700 Confocal Microscope and an Olympus IX71 (Olympus corporation, Tokyo, Japan). All immunohistochemistry slides were obtained as consecutive 40 μm slices. Images of consecutive basolateral amygdala (BLA) slices ranging from −1.0 mm to −2.3 mm were taken under a 20× objective by tile scans (3 × 4 tiles from aBLA and mBLA; 4 × 4 tiles for pBLA) coupled with z-stack. For pERK immunohistochemistry, images were obtained with a 40× objective at five different locations in the left and right DMS. Four of five locations were vertically aligned right next to the ventricle (the location positions were the same for all samples). Four brain slices were analyzed for each mouse.

Before analyzing c-Fos expression in the BLA, z-stack images were converted into 2D images by applying the Maximum Orthogonal Projection (MOP) in ZEN (blue edition) software. All images were analyzed by Meta Imaging Series® MetaMorph software 7.10.1.161. For identifying c-Fos expressing cells, the Count Nuclei (CN) functions with set values of minimum and maximum cell nucleus diameter was used to distinguish nucleus-localized c-Fos from accumulated noise (a consequence of MOP). Then, the c-Fos-expressing cells identified by CN in the BLA region were manually counted. The number of colocalized c-Fos+ cells (green) with retrograde tracer signal (red) from DMS was manually counted in ZEN software. In the DMS, pERK images were analyzed using the Integrated Morphometry Analysis (IMA) function using an inclusive threshold for both axon terminal (red) and pERK (green) puncta areas.

### In vitro electrophysiology

For slice preparations, mice were deeply anesthetized with a 1.25% Avertin solution (8 g of 2,2,2-Tribromoethanol and 5.1 mL of 2-methyl-2-butanol in 403 mL PBS, Sigma-Aldrich) and transcardially perfused with ice-cold dissection buffer (in mM): 180 sucrose, 2.5 KCl, 1.25 NaH_2_PO_4_, 25 NaHCO_3_, 11 glucose, 2 MgSO_4_, and 1 CaCl_2_ oxygenated with 95% O_2_/5% CO_2_); thereafter, mice were decapitated and their brains removed. Coronal brain slices (300 μm) were cut using a vibratome (VT 1000 S, Leica Microsystems) and allowed to recover for ≥15 min in artificial cerebrospinal fluid (ACSF) solution (in mM: 126 NaCl, 3 KCl, 1.25 NaH_2_PO_4_, 2 MgSO_4_, 2 CaCl_2_, 25 NaHCO_3_, and 10 glucose, which was continuously oxygenated with 95% O_2_/5% CO_2_) and later maintained at 32 °C. Slices were further incubated in ACSF (Room temperature, 22-25 °C) for ≥30 min before whole-cell voltage-clamp recordings.

For whole-cell voltage-clamp recordings, brain slices were placed in a recording chamber (room temperature, 22–25 °C) continuously perfused with oxygenated ACSF. The patch electrodes were pulled from standard-walled with filament borosilicate tubing (Standard glass capillaries, WPI; 5–6 MΩ resistance) filled with an intracellular solution (in mM: 130 mM Cs-methanesulfonate, 8 NaCl, 10 HEPES, 4 MgATP, 0.4 NaGTP, 0.5 EGTA and 5 QX-314 chloride [290 mOsm and pH 7.3]). After the break-through, ≥3 min was allowed for stabilization before starting with the recordings. oEPSCs were measured at the holding potential of −70 mV and +40 mV, from which AMPA and NMDA receptor-mediated currents were analyzed, respectively. Neurons with >20% change in series resistance were discarded.

All signals were amplified (MultiClamp 700B amplifier, Molecular Devices), low-pass filtered at 10 kHz, and acquired at 5 kHz using a data acquisition interface (ITC-18, HEKA Elektronik). Igor Pro software (WaveMetrics) was used for generating command signals, acquiring data, and data analysis.

We conducted whole-cell voltage-clamp experiments assuming that cells expressing tdTomato (tdT+) were dopamine receptor D1-expressing neurons (D1-MSNs) and those not expressing tdT (tdT-) were dopamine receptor D2-expressing neurons (D2-MSNs). To measure blue light stimulation of BLA-evoked oEPSC in D1- or D2-MSNs, 470-nm light was delivered to activate ChR2-expressing BLA axons, and oEPSCs were measured in either D1-or D2-MSNs. The blue light stimulation was delivered using a light-emitting diode (LED) (X-Cite, Lumen Dynamics) through ×40 water-immersion objective lens (Olympus, LUMPLFLN 40XW). The soma of the target cell was placed at the center of the illumination area while activating ChR2-expressing BLA axons. To measure the stimulus-response curve, a single light pulse (470 nm, 1 ms) at each light power (10, 15, 20, and 30% maximal light power, 15 mW) was delivered. For AMPA/NMDA ratio measurement, the peak amplitudes of the oEPSCs recorded at the holding potential of −70 mV (averaged over 5 repetitions) were used as AMPA receptor-mediated current. The mean oEPSC amplitude between 45–55 ms after the onset of blue light stimulation of BLA axons, recorded at the holding potential of +40 mV (averaged over 5 repetitions), was used as NMDA receptor-mediated current. The AMPA/NMDA ratio was calculated by dividing the AMPA receptor-mediated current by the NMDA receptor -mediated current. All AMPA receptor- and NMDA receptor-mediated currents were measured using the same light stimulation intensity (30% maximal light power, 15 mW). Igor Pro 6.37 software (WaveMetrics) was used for analyzing electrophysiology data.

### Western blotting

Brains were extracted immediately after sacrificing the mice and washed in HEPES buffer. All tissue preparation procedures took place under ice-cold conditions. The region from AP −1.5 mm to AP −0.5 mm from bregma was coronally dissected with a 3d printed brain matrix (https://www.thingiverse.com/thing:4339995) and the bilateral dorsomedial striata were collected into a glass Teflon homogenizer. The collected tissue was homogenized in 200 μL of 0.32 M HEPES-buffered sucrose solution containing a protease inhibitor cocktail (1:200; Biomax, BPI0001), 50 mM NaF, and 1 mM NaVO_4_. The homogenate was centrifuged at 1000 × *g* for 5 min at 4 °C. Then, the pellet was resuspended with 100 μL of homogenization buffer in a glass Teflon homogenizer and homogenized. This supernatant was combined with the previous supernatant and stored at −80 °C until protein quantification.

Protein quantification was conducted using a BCA protein assay (Thermo Scientific). The same amount of protein was mixed with a sample buffer containing β-mercaptoethanol and denatured by boiling for 10 min at 95 °C. Samples were electrophoretically separated by 8% SDS-PAGE and transferred onto a PVDF membrane, which was blocked with 5% BSA or 5% skim milk in 1X PBS for 1 h. After blocking, the membrane was incubated with primary antibodies overnight (16–18 h) at 4 °C. Each antibody was diluted with PBST (0.05% Tween-20) [anti-Phospho-p44/42 MAPK (1:1000, Cell Signaling, 9101), anti-p44/42 MAPK (1:1000, Cell Signaling, 9102)]. Anti-rabbit secondary antibody conjugated with horseradish peroxidase (1:5000, GenDepot, SA202) was used for detecting the primary antibodies by incubating the membrane for 1 h at room temperature. ECL reagents (Cytiva, RPN2232) were incubated with the membrane for 2 min and bands were imaged with a Las-4000 mini (ImageQuant). Membranes were stripped, re-probed with another antibodies, imaged with same procedure aforementioned.

### Statistics

Data were analyzed using one-way or two-way analysis of variance (ANOVA), Student’s paired *t-*test or unpaired t-test. We used a Shapiro–Wilk test for testing normal distribution. Where appropriate, a repeated measures ANOVA was used or an ANOVA followed by post hoc Student’s *t*-test or Tukey’s test, Tukey-Kramer test or Sidak’s multiple comparisons test. If data did not follow a normal distribution, Friedman test, Mann–Whitney test, Wilcoxon matched-pairs signed rank test, or Kruskal–Wallis test followed by post hoc Dunn’s multiple comparisons test were used. For comparing cumulative probability data, we used the Kolmogorov–Smirnov test. Statistical analyses were performed using SPSS 27 (IBM). A *p* value < 0.05 was considered statistically significant. *P < 0.05, **P < 0.01, ***P < 0.001, ****P < 0.0001.

### Reporting summary

Further information on research design is available in the Nature Portfolio Reporting Summary linked to this article.

### Supplementary information

Supplementary Information

Peer Review File

Description of Additional Supplementary Files

Supplementary Movie 1

Supplementary Movie 2

Supplementary Movie 3

Reporting Summary

### Source data

Source Data file

## Data Availability

All image data used in this study have been deposited in figshare (10.6084/m9.figshare.24669447.v1). Source video data are available from the corresponding author upon request. Source data are provided with this paper.
