# Supplementary material for: Persistent enhancement of basolateral amygdala-dorsomedial striatum synapses causes compulsive-like behaviors in mice
Source: Nat Commun. 2024 Jan 8;15:219. doi: 10.1038/s41467-023-44322-8 (PMC10774417; doi:10.1038/s41467-023-44322-8)
Supplement: Supplementary file 1 — Supplementary Information [file 41467_2023_44322_MOESM1_ESM.pdf]

SUPPLEMENTARY INFORMATION

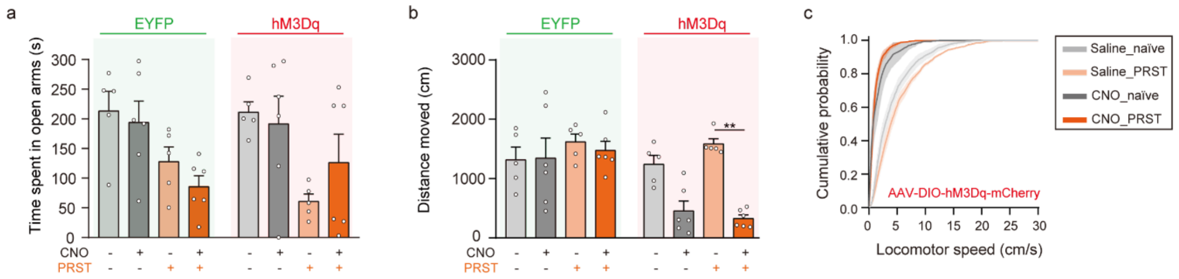

Supplementary Fig. 1. Specific activation of D2-MSNs in the dorsal striatum decreased repetitive behavior. **a-b** Behaviors on the EPM after specific activation of D2-MSNs by i.p. CNO injection in D2-CRE::AAV-DIO-hM3Dq mice. ($\text{EYFP}_{\text{Saline}}$, $n = 5$; EYFP_{CNO}, $n = 6$; $\text{hM3Dq}_{\text{Saline}}$, $n = 5$; $\text{hM3Dq}_{\text{CNO}}$, $n = 6$). A two-way ANOVA found a significant effect of stress (PRST) on time spent in the open arms in the EYFP group (**a**), and a Kruskal–Wallis test with *post hoc* Dunn's multiple comparisons test revealed a significance for a total distance traveled in EPM in hM3Dq-expressing mice (**b**) ($H = 15.49$, $P = 0.0014$). **c** Cumulative probability plot for the locomotor speed in hM3Dq-expressing mice. A Kolmogorov–Smirnov test was used to analyze data (Saline_PRST *vs* CNO_PRST, $P < 0.0001$; CNO_naive *vs* CNO_PRST, $P < 0.0001$). Data are represented as mean ± SEM.

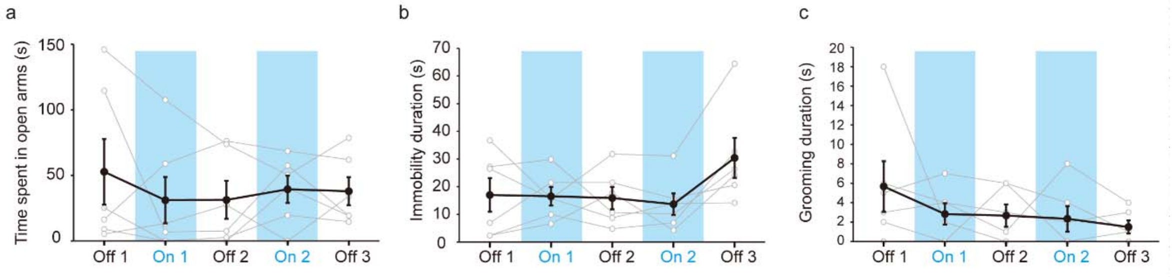

Supplementary Fig. 2. Optical stimulation in the EYFP group. **a-c** Behavioral responses induced by optical stimulation of the BLA-DMS circuit in EYFP controls ($n = 6$). No significant effect of optical stimulation was observed either on time spent in open arms (**a**), immobility (**b**), or grooming duration (**c**). Data are represented as mean ± SEM.

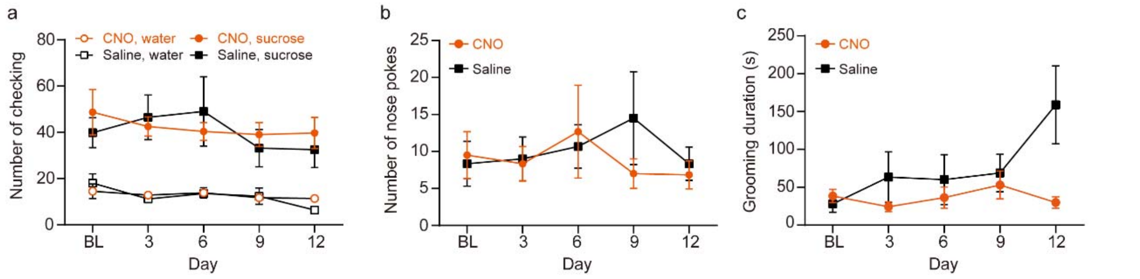

Supplementary Fig. 3. Effect of chronic CNO injections (i.p.) in naive mice. **a** Number of compulsive checking occurrences in sucrose well or water well. **b** Number of repetitive nose pokes into any hole. **c** Durations of compulsive grooming. (Saline, $n = 6$; CNO, $n = 6$). Data are represented as mean ± SEM.

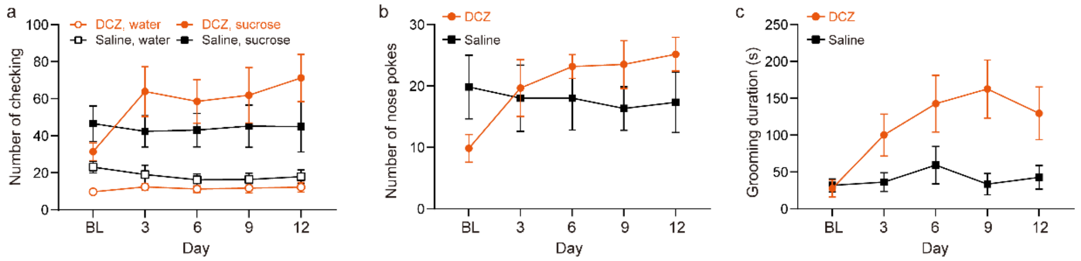

Supplementary Fig. 4. Chronic activation effects of the BLA-DMS circuit with DCZ. a Number of repetitive checking (visits) to water or sucrose wells. A two-way repeated measures ANOVA revealed that a significant interaction between the effects of time and treatment (DCZ) on checking on sucrose well ($F_{4, 40} = 8.294$, $P < 0.0001$). **b** Number of repetitive nose pokes into random holes. A two-way repeated measures ANOVA revealed a significant interaction between the effects of time and treatment (DCZ) on nose pokes ($F_{4, 40} = 3.188$, $P = 0.0230$). **c** Duration of excessive grooming. A two-way repeated measures ANOVA revealed a significant interaction between the effects of time and treatment (DCZ) on grooming duration ($F_{4, 40} = 3.016$, $P = 0.0289$). (Saline, $n = 6$; DCZ, $n = 6$). Data are represented as mean ± SEM.

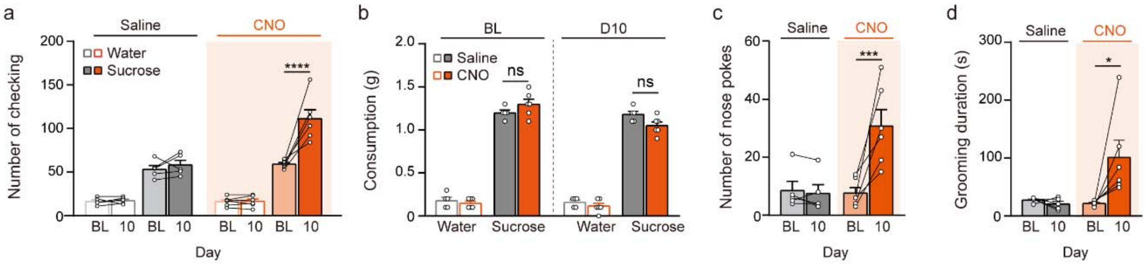

Supplementary Fig. 5. Compulsive-like behaviors of D1-tdTomato mice used for electrophysiological recording. a-d Daily chronic CNO (1 mg/kg) injections (i.p.) were performed for 10 days after 3 days of habituation. D1-tdTomato mice were injected with AAV-DIO-hM3Dq in the BLA and with retroAAV-Cre in the DMS (Sal, $n = 5$; CNO, $n = 6$). The number of checking to sucrose well was significantly increased in the CNO group (**a**) while the amount of sucrose or water consumption did not change in both groups (**b**). The number of repetitive nose pokes (**c**) and duration of grooming (**d**) also significantly increased in the CNO-treated group. Data were analyzed with a two-way repeated measures ANOVA with *post hoc* Sidak's multiple comparisons test. Data are represented as mean ± SEM.

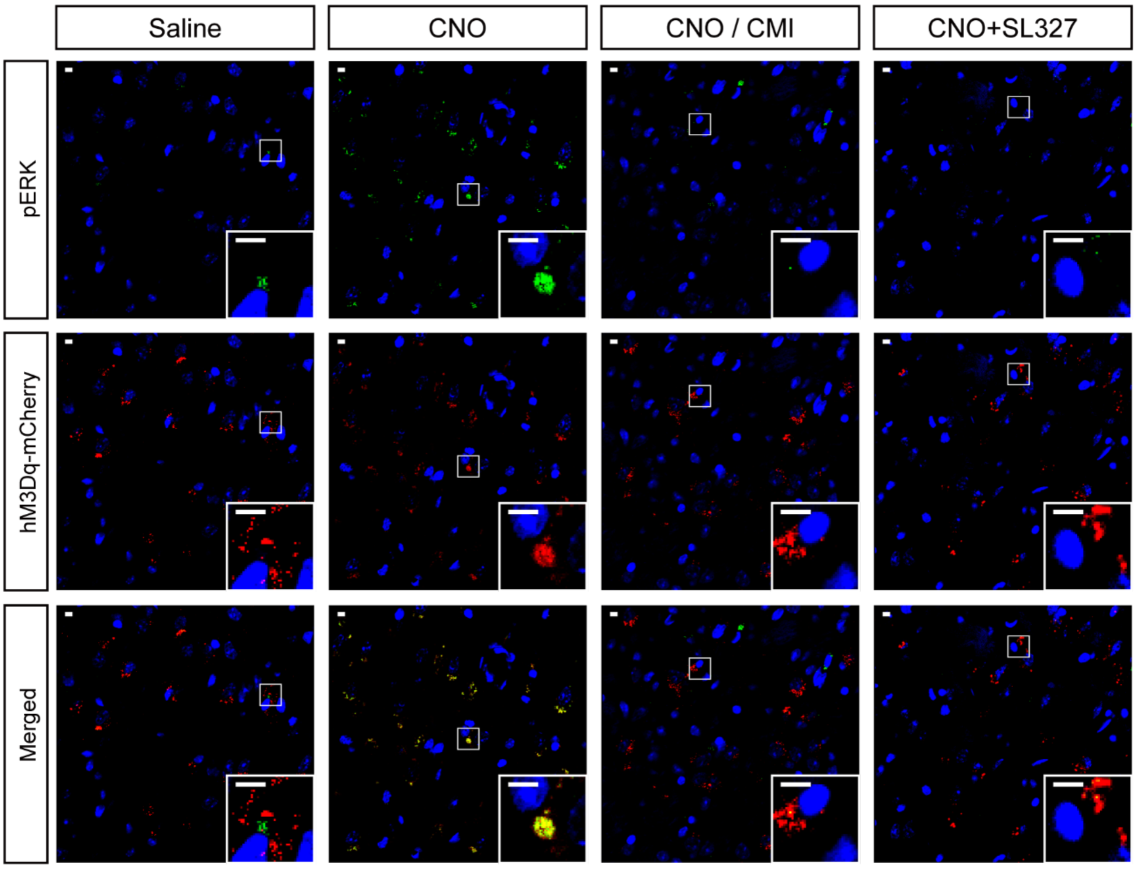

Supplementary Fig. 6. Chronic chemogenetic activation of the BLA-DMS circuit increases ERK phosphorylation level within the BLA. Representative images of pERK, hM3Dq-mCherry, and merged signals from four experimental groups (D13–15: Saline, CNO, CNO+SL327; D30–33: CNO/CMI). Scale bars indicate 50 μm.

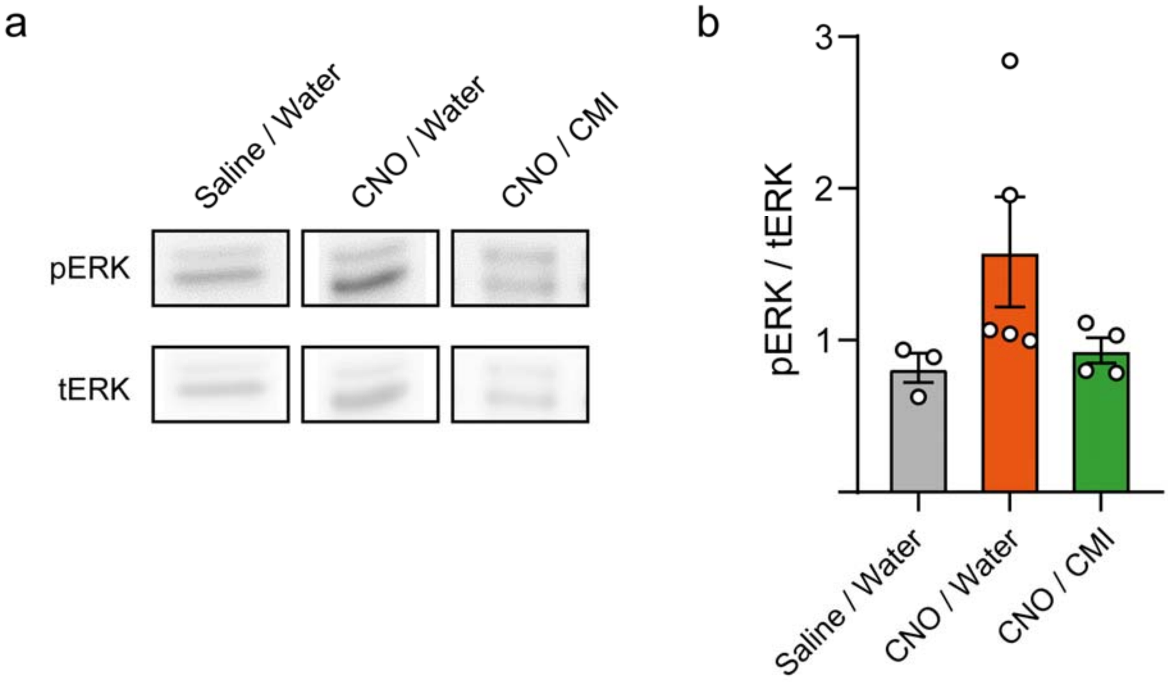

Supplementary Fig. 7. Immunoblotting assay results of chronic BLA-DMS activation. a Representative images of immunoblotting assay. Tissue samples (DMS) were taken after two weeks of CMI administration. **b** Quantification of immunoblotting assay. Data were analyzed with Kruskal–Wallis test with *post hoc* Dunn's multiple comparisons test ($H = 5.426$, $P = 0.0566$). Data are represented as mean ± SEM.

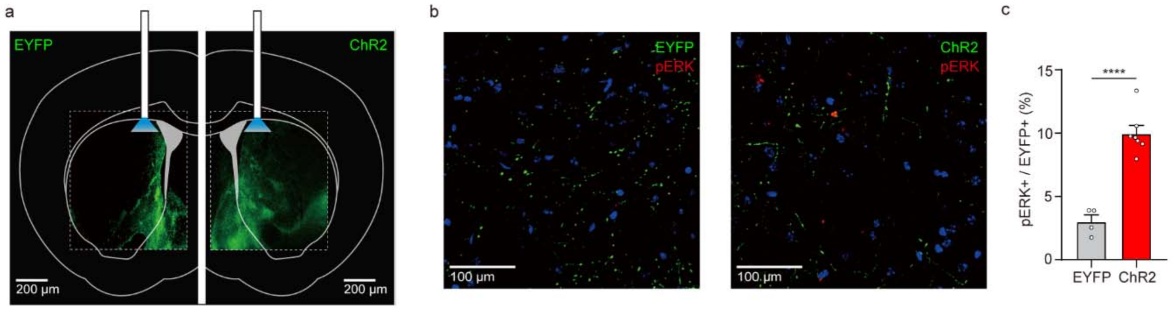

Supplementary Fig. 8. Repetitive optical stimulations of the BLA-DMS circuit induce the phosphorylation of ERK colocalized to BLA axons. a Diagram showing the position of an implanted optic fiber with representative images of projections from BLA neurons expressing ChR2-EYFP (left) or EYFP (right) in the DMS. **b** Representative images showing phosphorylated ERK (red) and BLA axons (green) in the DMS after repetitive optical stimulations (5 days) of the BLA-DMS circuit. **c** Level of pERK colocalizing with BLA axons in the DMS. Data were analyzed with an unpaired *t*-test, ($t = 7.393$, $df = 9$, $P < 0.0001$; ChR2, $n = 7$ mice; EYFP, $n = 4$ mice). Data are represented as mean ± SEM.

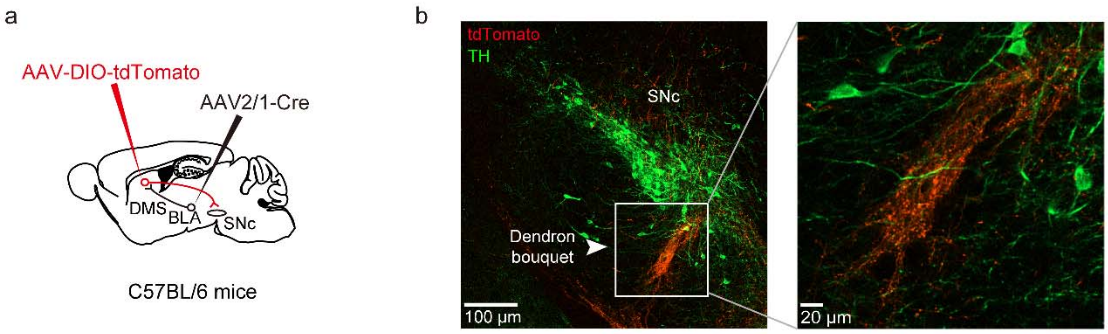

Supplementary Fig. 9. DMS neurons receiving BLA inputs send axons to the SNc. a Experimental scheme of transsynaptic tracing to identify the projection targets of DMS neurons that receive the BLA inputs. **b** Representative image of the projected axons from DMS neurons receiving the BLA input in the SNc. Note that the axons of DMS neurons overlap with a dendron-bouquet structure.

Lane	1	2	3	4	5	6	7	8
	DMS S1 (lysate)				DMS P2 (crude synaptosome)			
	Saline	CNO	CNO	CNO	Saline	CNO	CNO	CNO
	Water	CMI	CMI	Water	Water	CMI	CMI	CMI

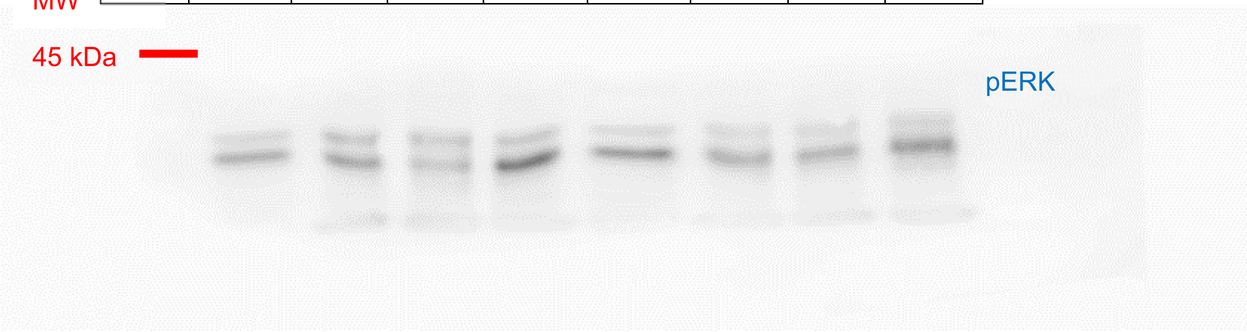

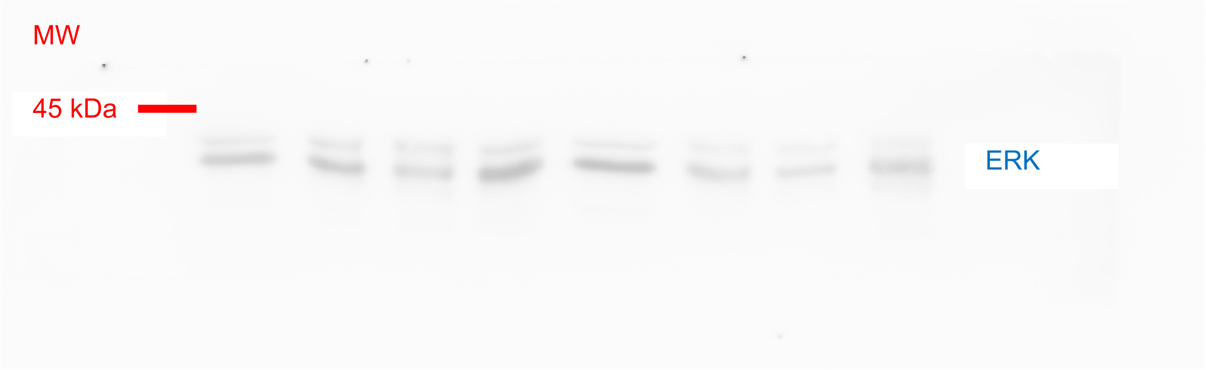

Blot 1.

Related to Extended figure. .

Lane 1, 3, 4 images were cropped and quantificated for figure.

Lane	1	2	3	4	5	6	7	8
	S1 (lysate)				P2 (crude synaptosome)			
	DMS CNO Water	DLS CNO Water	DMS CNO CMI	DLS CNO CMI	DMS CNO Water	DLS CNO Water	DMS CNO CMI	DLS CNO CMI

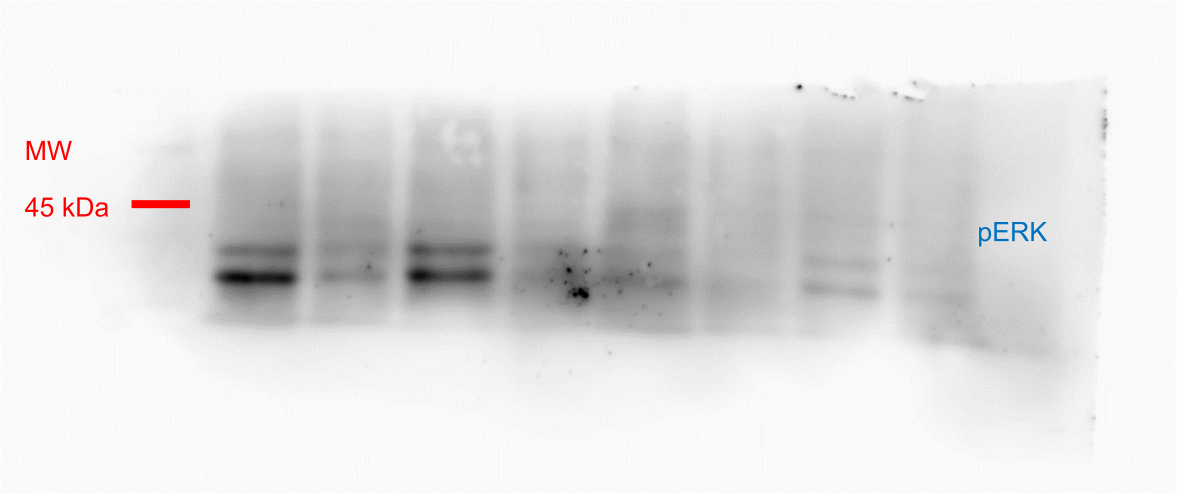

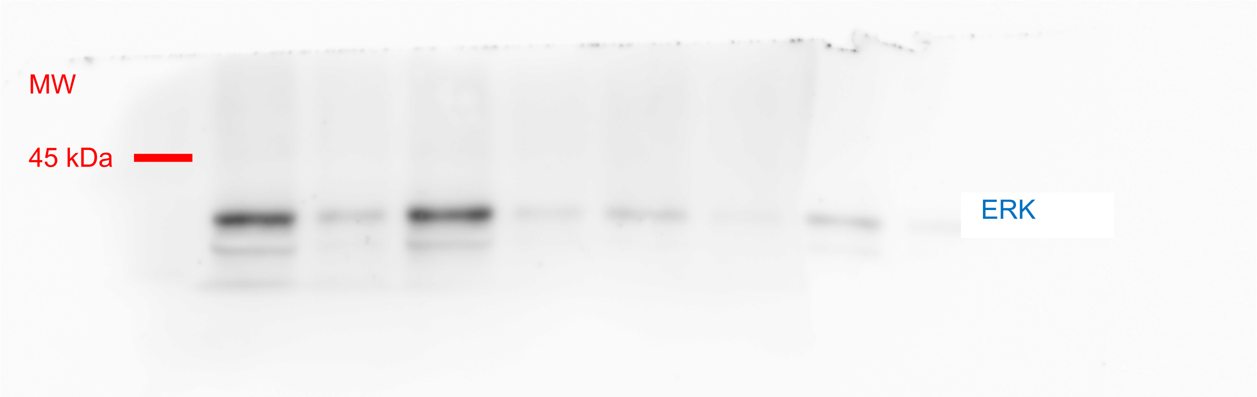

Blot 2.

Lane 1, 3 were quantificated for figure.

Lane	1	2	3	4	5	6	7	8
	S1 (lysate)				P2 (crude synaptosome)			
	DMS CNO Water	DLS CNO Water	DMS CNO CMI	DLS CNO CMI	DMS CNO Water	DLS CNO Water	DMS CNO CMI	DLS CNO CMI

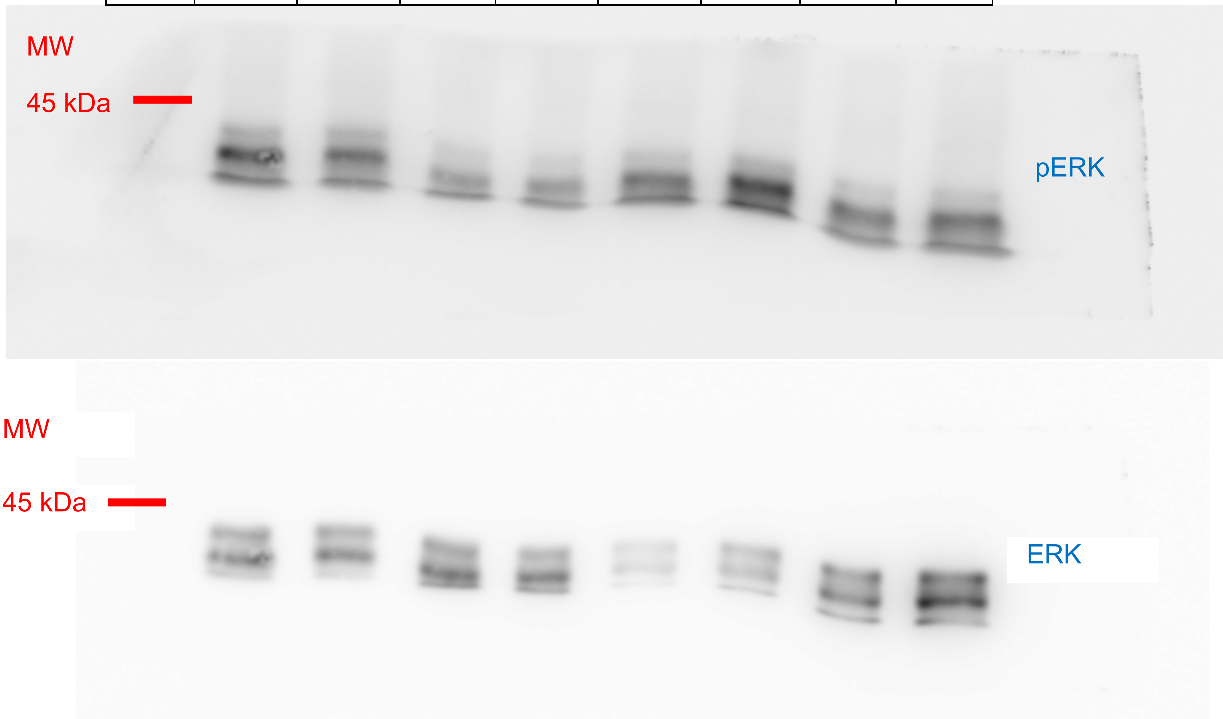

Blot 3.

Lane 1, 3 were quantificated for figure.

Lane	1	2	3	4	5	6	7	8
	S1 (lysate)				P2 (crude synaptosome)			
	DMS CNO Water	DLS CNO Water	DMS CNO CMI	DLS CNO CMI	DMS CNO Water	DLS CNO Water	DMS CNO CMI	DLS CNO CMI

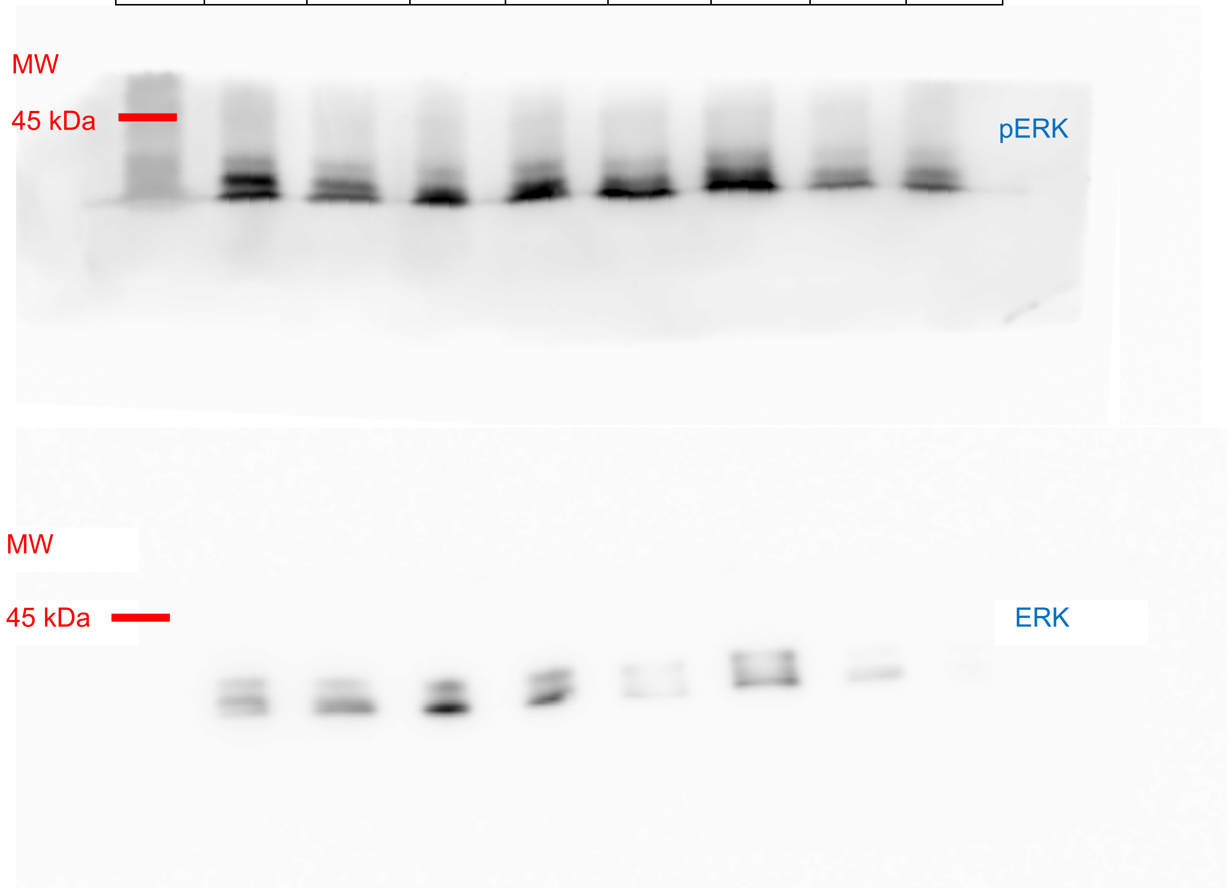

Blot 4.

Lane 1, 3 were quantificated for figure.

Lane	1	2	3	4
	S1 (lysate)			
	DMS CNO Water	DMS Saline Water	DMS Saline Water	DMS Saline Water

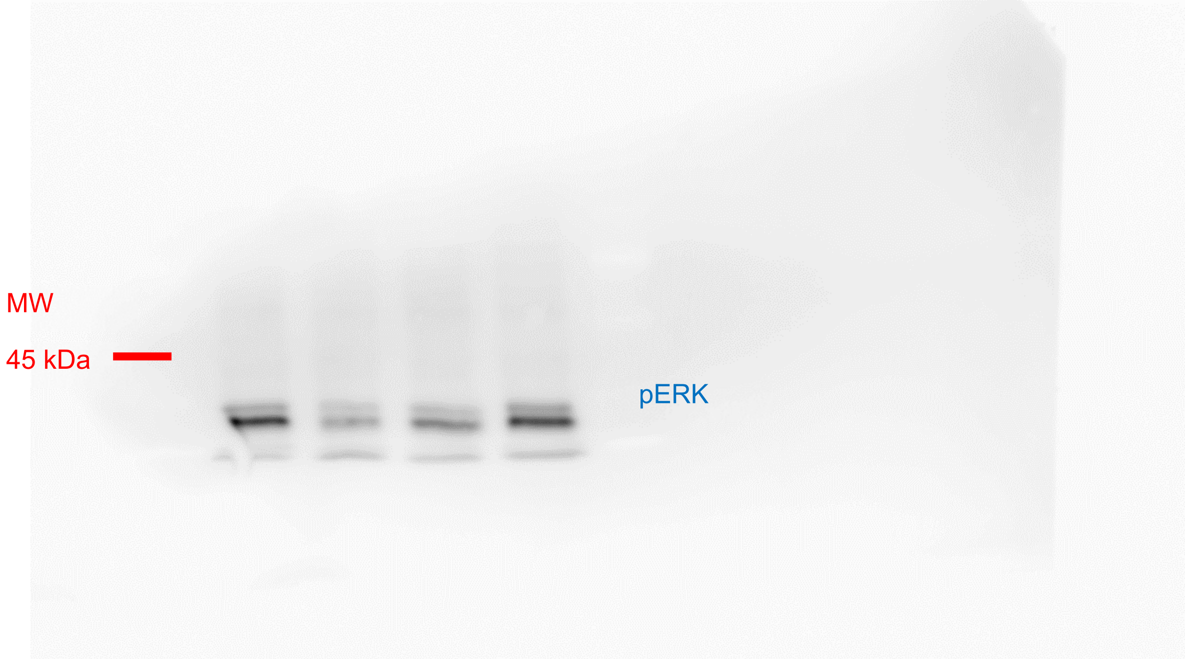

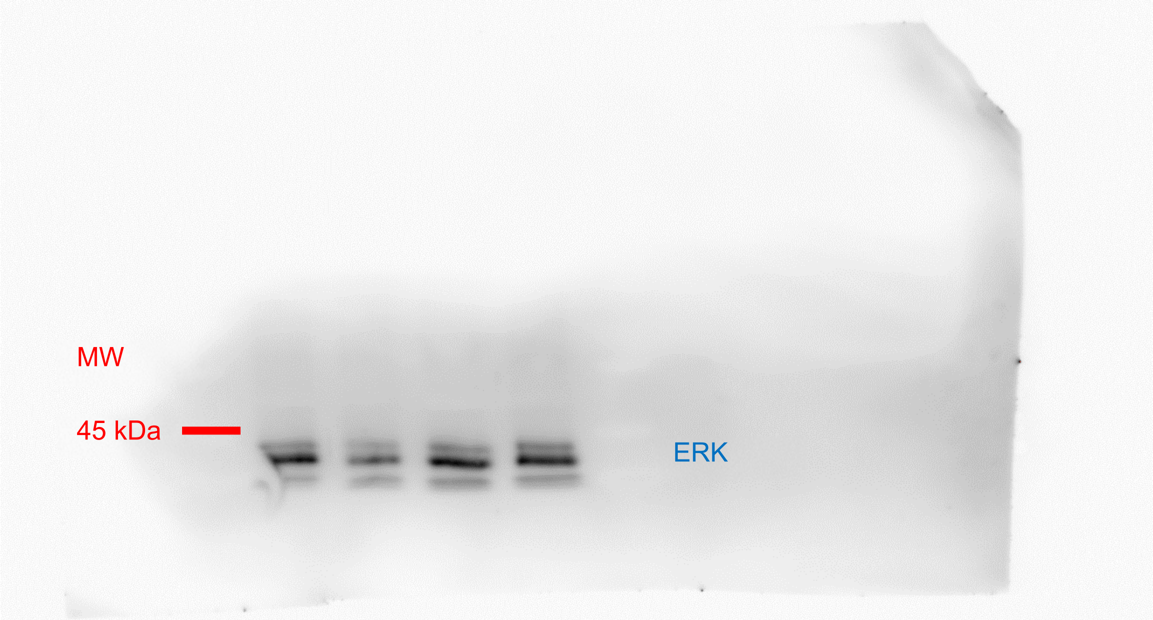

Blot 5.

Lane 2 is same sample with Lane 1 in Blot 1.

Lane 1, 3, 4 were quantificated for figure.
