# Supplementary material for: Persistent enhancement of basolateral amygdala-dorsomedial striatum synapses causes compulsive-like behaviors in mice
Source: Nat Commun. 2024 Jan 8;15:219. doi: 10.1038/s41467-023-44322-8 (PMC10774417; doi:10.1038/s41467-023-44322-8)
Supplement: Supplementary file 3 — Description of Additional Supplementary Files [file 41467_2023_44322_MOESM3_ESM.pdf]

<u>Description of Additional Supplementary Files</u>

Supplementary Movies

Supplementary Movie 1. Exemplary movie for anxious behavior on EPM with optical stimulation.
Supplementary Movie 2. Exemplary movie for compulsive-like behaviors on holeboard test.
Supplementary Movie 3. Exemplary movie for hoarding test.
