# Supplementary material for: Persistent enhancement of basolateral amygdala-dorsomedial striatum synapses causes compulsive-like behaviors in mice
Source: Nat Commun. 2024 Jan 8;15:219. doi: 10.1038/s41467-023-44322-8 (PMC10774417; doi:10.1038/s41467-023-44322-8)
Supplement: Supplementary file 7 — Reporting Summary [file 41467_2023_44322_MOESM7_ESM.pdf]

Corresponding author(s): Bong-June Yoon
Last updated by author(s): Nov 29, 2023

Reporting Summary

Nature Portfolio wishes to improve the reproducibility of the work that we publish. This form provides structure for consistency and transparency in reporting. For further information on Nature Portfolio policies, see our Editorial Policies and the Editorial Policy Checklist.

Statistics

For all statistical analyses, confirm that the following items are present in the figure legend, table legend, main text, or Methods section.

n/a	Confirmed	
☐	☒	The exact sample size (*n*) for each experimental group/condition, given as a discrete number and unit of measurement
☐	☒	A statement on whether measurements were taken from distinct samples or whether the same sample was measured repeatedly
☐	☒	The statistical test(s) used AND whether they are one- or two-sided<br>*Only common tests should be described solely by name; describe more complex techniques in the Methods section.*
☐	☒	A description of all covariates tested
☐	☒	A description of any assumptions or corrections, such as tests of normality and adjustment for multiple comparisons
☐	☒	A full description of the statistical parameters including central tendency (e.g. means) or other basic estimates (e.g. regression coefficient) AND variation (e.g. standard deviation) or associated estimates of uncertainty (e.g. confidence intervals)
☐	☒	For null hypothesis testing, the test statistic (e.g. *F*, *t*, *r*) with confidence intervals, effect sizes, degrees of freedom and *P* value noted<br>*Give P values as exact values whenever suitable.*
☒	☐	For Bayesian analysis, information on the choice of priors and Markov chain Monte Carlo settings
☒	☐	For hierarchical and complex designs, identification of the appropriate level for tests and full reporting of outcomes
☒	☐	Estimates of effect sizes (e.g. Cohen's *d*, Pearson's *r*), indicating how they were calculated

Our web collection on statistics for biologists contains articles on many of the points above.

Software and code

Policy information about availability of computer code

Data collection	Electrohysiology data were collected using Igor Pro 6.37 software (WaveMetrics)
Data analysis	EthoVision XT 11.5 software (Noldus, Wageningen, Netherlands) was used for analyzing behavior. Meta Imaging Series® MetaMorph 7.10.1.161 was used for analyzing image. Igor Pro 6.37 software (WaveMetrics) was used for analyzing electrophysiology data.

For manuscripts utilizing custom algorithms or software that are central to the research but not yet described in published literature, software must be made available to editors and reviewers. We strongly encourage code deposition in a community repository (e.g. GitHub). See the Nature Portfolio guidelines for submitting code & software for further information.

Data

Policy information about availability of data

All manuscripts must include a data availability statement. This statement should provide the following information, where applicable:

- Accession codes, unique identifiers, or web links for publicly available datasets
- A description of any restrictions on data availability
- For clinical datasets or third party data, please ensure that the statement adheres to our policy

Data supporting the findings of this study are available within the supplementary information and source data files provided with this paper. Source video and image data are available from the corresponding author upon request.

Research involving human participants, their data, or biological material

Policy information about studies with human participants or human data. See also policy information about sex, gender (identity/presentation), and sexual orientation and race, ethnicity and racism.

Reporting on sex and gender	N/A
Reporting on race, ethnicity, or other socially relevant groupings	N/A
Population characteristics	N/A
Recruitment	N/A
Ethics oversight	N/A

Note that full information on the approval of the study protocol must also be provided in the manuscript.

Field-specific reporting

Please select the one below that is the best fit for your research. If you are not sure, read the appropriate sections before making your selection.

☒ Life sciences ☐ Behavioural & social sciences ☐ Ecological, evolutionary & environmental sciences

For a reference copy of the document with all sections, see nature.com/documents/nr-reporting-summary-flat.pdf

Life sciences study design

All studies must disclose on these points even when the disclosure is negative.

Sample size	No sample-size predetermination method was used, but our sample sizes are similar to those reported in previous publications. (https://doi.org/10.1038/s41380-023-02019-w, https://doi.org/10.1038/s41386-021-01161-9)
Data exclusions	Animals were excluded only if viral injection or fiber implantation was misplaced.
Replication	All representative data were repeated at least three times with similar results.
Randomization	Mice were randomly allocated to distinct group.
Blinding	Researchers were blinded to the group allocation of animals during data collection and data analyses for all the experiments in our study.

Reporting for specific materials, systems and methods

We require information from authors about some types of materials, experimental systems and methods used in many studies. Here, indicate whether each material, system or method listed is relevant to your study. If you are not sure if a list item applies to your research, read the appropriate section before selecting a response.

Materials & experimental systems

n/a	Involved in the study	
☐	☒	Antibodies
☐	☒	Eukaryotic cell lines
☒	☐	Palaeontology and archaeology
☐	☒	Animals and other organisms
☒	☐	Clinical data
☒	☐	Dual use research of concern
☒	☐	Plants

Methods

n/a	Involved in the study	
☒	☐	ChIP-seq
☒	☐	Flow cytometry
☒	☐	MRI-based neuroimaging

Antibodies

Antibodies used	Primary antibodyies<br>- anti-c-Fos (1:2000, abcam, ab190289)<br>- anti-Mu opioid receptor (1:400, abcam, ab10275)

- anti-Phospho-p44/42 MAPK (1:1,000, Cell signaling, 9101)
- anti-p44/42 MAPK (1:1,000, Cell signaling, 9102)
- anti-Tyrosine Hydroxylase (1:500, Millipore, ab152)

Secondary antibodies
- Alexa Fluor 488 donkey anti-rabbit (1:400, Invitrogen, A-21206)
- Alexa Fluor 594 donkey anti-rabbit (1:400, Invitrogen, A-21207)
- Goat anti-Rabbit IgG(H+L)-HRP (1:5,000, GenDepot, SA202)

Validation

Antibodies were validated by the manufacturer and/or studies cited on the company's website.

- anti-c-Fos (abcam, ab190289) : https://www.abcam.com/products/primary-antibodies/c-fos-antibody-bsa-free-ab190289.html
- anti-Mu opioid receptor (abcam, ab10275) : https://www.abcam.com/products/primary-antibodies/mu-opioid-receptor-antibody-ab10275.html
- anti-Phospho-p44/42 MAPK (Cell signaling, 9101) : https://www.cellsignal.com/products/primary-antibodies/phospho-p44-42-mapk-erk1-2-thr202-tyr204-antibody/9101
- anti-p44/42 MAPK (Cell signaling, 9102) : https://www.cellsignal.jp/products/primary-antibodies/p44-42-mapk-erk1-2-antibody/9102?N=4294956287&Ntt=erk&fromPage=plp
- anti-Tyrosine Hydroxylase (Millipore, ab152) : https://www.merckmillipore.com/KR/ko/product/Anti-Tyrosine-Hydroxylase-Antibody,MM_NF-AB152

Eukaryotic cell lines

Policy information about cell lines and Sex and Gender in Research

Cell line source(s)	AAV-293 cells were purchased from Agilient (AAV Helper-Free system, Cat. #240071)
Authentication	The cell line was not authenticated.
Mycoplasma contamination	The cell line was tested negative for mycoplasma contamination.
Commonly misidentified lines (See ICLAC register)	No commonly misidentified cell lines were used in this study.

Animals and other research organisms

Policy information about studies involving animals; ARRIVE guidelines recommended for reporting animal research, and Sex and Gender in Research

Laboratory animals

Transgenic mice used in this study [Drd1a-tdTomato (B6.Cg-Tg(Drd1a-tdTomato)6Calak/J), Drd1a-EGFP (Tg(Drd1-EGFP) × 60Gsat/Mmmh), Drd2-EGFP (Tg(Drd2-EGFP)S118Gsat/Mmnc), and Drd1a-CRE (B6.FVB(Cg)-Tg(Drd1-cre)EY217Gsat/Mmucd), Drd2a-CRE (B6.FVB(Cg)–Tg(Drd2-cre) ER44Gsat/Mmucd] were purchased from the Jackson Laboratory and Mutant Mouse Resource and Research Center. C57BL/N mice and Crl:CD(SD) rats were purchased from Orient Bio.
Animals were maintained under controlled temperature (23°C) and humidity conditions (50%) with a 12-h light-dark cycle (light on at 7 AM). Food and water were available ad libitum except for water deprivation (overnight) experiment for c-Fos measurement.
All mice used in this study were 8-20 weeks old and rats were over 12 weeks old.

Wild animals	This study did not involve wild animals.
Reporting on sex	Both male and female mice were used for chemogenetic activation experiment described in Figure 1 and electrophysiological study for confirming connectivity in Figure 2. Only male mice were used in all other experiments to avoid possible complications due to gender differences in OCD (DOI: 10.1007/s11920-019-1015-2).
Field-collected samples	No field-collected samples were used in this study.
Ethics oversight	All experimental procedures in this study were approved by the Korea University Institutional Animal Care and Use Committees.

Note that full information on the approval of the study protocol must also be provided in the manuscript.
