# Supplementary material for: Persistent enhancement of basolateral amygdala-dorsomedial striatum synapses causes compulsive-like behaviors in mice
Source: Nat Commun. 2024 Jan 8;15:219. doi: 10.1038/s41467-023-44322-8 (PMC10774417; doi:10.1038/s41467-023-44322-8)
Supplement: Supplementary file 2 — Peer Review File [file 41467_2023_44322_MOESM2_ESM.pdf]

nature portfolio

Peer Review File

Persistent enhancement of basolateral amygdala-dorsomedial striatum synapses causes compulsive-like behaviors in mice

Open Access This file is licensed under a Creative Commons Attribution 4.0 International License, which permits use, sharing, adaptation, distribution and reproduction in any medium or format, as long as you give appropriate credit to the original author(s) and the source, provide a link to the Creative Commons license, and indicate if changes were made. In the cases where the authors are anonymous, such as is the case for the reports of anonymous peer reviewers, author attribution should be to 'Anonymous Referee' followed by a clear attribution to the source work. The images or other third party material in this file are included in the article's Creative Commons license, unless indicated otherwise in a credit line to the material. If material is not included in the article's Creative Commons license and your intended use is not permitted by statutory regulation or exceeds the permitted use, you will need to obtain permission directly from the copyright holder. To view a copy of this license, visit http://creativecommons.org/licenses/by/4.0/.

Editorial Note: This manuscript has been previously reviewed at another journal that is not operating a transparent peer review scheme. This document only contains reviewer comments and rebuttal letters for versions considered at Nature Communications.

REVIEWERS' COMMENTS

Reviewer #3 (Remarks to the Author):

This excellent paper analyzes projections from the basolateral amygdala to the dorsomedial striatum in mice and characterizes the consequences of stimulation of this circuitry, which produces repetitive ('compulsive-like') behavior of plausible relevance to OCD and other neuropsychiatric disorders. My primary concern with the original submission was the framing, which was based on a simplistic and inappropriate equation of mouse behaviors with human OCD. This has been fully addressed in this revision, which appropriately reframes the study as an elegant circuit neuroscience analysis of a relatively neglected projection, while maintaining appropriate discussion of the potential relevance to OCD and other neuropsychiatric disorders characterized by compulsivity as a motivating context. With this reframing the paper is simplified and emerges as an elegant and rigorous study eminently worthy of rapid publication. The relatively minor additional issues that I and the other reviewers of the earlier submission pointed out have been appropriately addressed; I have no further suggestions.
